# Towards unambiguous reporting of complications related to deep brain stimulation surgery: A retrospective single-center analysis and systematic review of the literature

**DOI:** 10.1371/journal.pone.0198529

**Published:** 2018-08-02

**Authors:** Katja Engel, Torge Huckhagel, Alessandro Gulberti, Monika Pötter-Nerger, Eik Vettorazzi, Ute Hidding, Chi-un Choe, Simone Zittel, Hanna Braaß, Peter Ludewig, Miriam Schaper, Kara Krajewski, Christian Oehlwein, Katrin Mittmann, Andreas K. Engel, Christian Gerloff, Manfred Westphal, Christian K. E. Moll, Carsten Buhmann, Johannes A. Köppen, Wolfgang Hamel

**Affiliations:** 1 Klinik für Neurochirurgie, Universitätsklinikum Hamburg-Eppendorf, Hamburg, Germany; 2 Institut für Neurophysiologie & Pathophysiologie, Universitätsklinikum Hamburg-Eppendorf, Hamburg, Germany; 3 Klinik für Neurologie, Universitätsklinikum Hamburg-Eppendorf, Hamburg, Germany; 4 Institut für Medizinische Biometrie und Epidemiologie, Universitätsklinikum Hamburg-Eppendorf, Hamburg, Germany; 5 Praxis für Neurologie, Gera, Germany; University of Pennsylvania Perelman School of Medicine, UNITED STATES

## Abstract

**Background and objective:**

To determine rates of adverse events (AEs) related to deep brain stimulation (DBS) surgery or implanted devices from a large series from a single institution. Sound comparisons with the literature require the definition of unambiguous categories, since there is no consensus on the reporting of such AEs.

**Patients and methods:**

123 consecutive patients (median age 63 yrs; female 45.5%) treated with DBS in the subthalamic nucleus (78 patients), ventrolateral thalamus (24), internal pallidum (20), and centre médian-parafascicular nucleus (1) were analyzed retrospectively. Both mean and median follow-up time was 4.7 years (578 patient-years). AEs were assessed according to three unambiguous categories: (i) hemorrhages including other intracranial complications because these might lead to neurological deficits or death, (ii) infections and similar AEs necessitating the explantation of hardware components as this results in the interruption of DBS therapy, and (iii) lead revisions for various reasons since this involves an additional intracranial procedure. For a systematic review of the literature AE rates were calculated based on primary data presented in 103 publications. Heterogeneity between studies was assessed with the I^2^ statistic and analyzed further by a random effects meta-regression. Publication bias was analyzed with funnel plots.

**Results:**

Surgery- or hardware-related AEs (23) affected 18 of 123 patients (14.6%) and resolved without permanent sequelae in all instances. In 2 patients (1.6%), small hemorrhages in the striatum were associated with transient neurological deficits. In 4 patients (3.3%; 0.7% per patient-year) impulse generators were removed due to infection. In 2 patients electrodes were revised (1.6%; 0.3% per patient-year). There was no lead migration or surgical revision because of lead misplacement. Age was not statistically significant different (p>0.05) between patients affected by AEs or not. AE rates did not decline over time and similar incidences were found among all patients (423) implanted with DBS systems at our institution until December 2016. A systematic literature review revealed that exact AE rates could not be determined from many studies, which could not be attributed to study designs. Average rates for intracranial complications were 3.8% among studies (per-study analysis) and 3.4% for pooled analysis of patients from different studies (per-patient analysis). Annual hardware removal rates were 3.6 and 2.4% for per-study and per-patient analysis, respectively, and lead revision rates were 4.1 and 2.6%, respectively. There was significant heterogeneity between studies (I^2^ ranged between 77% and 91% for the three categories; p< 0.0001). For hardware removal heterogeneity (I^2^ = 87.4%) was reduced by taking study size (p< 0.0001) and publication year (p< 0.01) into account, although a significant degree of heterogeneity remained (I^2^ = 80.0%; p< 0.0001). Based on comparisons with health care-related databases there appears to be publication bias with lower rates for hardware-related AEs in published patient cohorts.

**Conclusions:**

The proposed categories are suited for an unequivocal assessment of AEs even in a retrospective manner and useful for benchmarking. AE rates in the present cohorts from our institution compare favorable with the literature.

## Introduction

Deep brain stimulation (DBS) is one of the most effective treatment modalities for patients suffering from movement disorders [1–7]. It is interesting to note that despite many technical advances and refinements, the overall efficacy of DBS has remained constant over the last few decades. Taking this into consideration, for properly selected patients the actual margin of the overall clinical benefit from DBS therapy will be related to AEs.

DBS surgery- and hardware-related AEs are more obvious than many neurological and psychiatric AEs facilitating the collection and rating (i.e., severity, reversibility and relatedness to DBS surgery or implanted hardware) of such AEs. However, despite the utilization of common categories, such as infections or hemorrhages, inconsistent reporting has hampered straightforward comparisons between studies. Whereas broad categories (e.g., infection of all kinds) may cover non-severe AEs (e.g., superficial wound healing abnormalities), narrowly defined categories (e.g., erosion) result in the dispersal of AE rates. A multitude of categories will be unsuited for benchmarking purposes. It is unlikely that the required information could be extracted from all publications. In addition, too detailed rates may confuse patients.

From a patient’s perspective, the risk of AEs should be based on subjects but not implanted hardware. For example, calculations based on electrodes result in the dilution of AEs rates as most patients become implanted with a bilateral DBS system. AE rates based on electrodes rather serve to evaluate the performance of hardware components. In addition, follow-up time is crucial to assess the cumulative risk for hardware-related AEs, which may occur even years after surgery. Aggregate follow-up of patient cohorts in patient-years can be deducted with ease from most studies (number of patients * mean follow-up) and used for comparison.

In the present study we provide a detailed retrospective analysis of surgery- and hardware-related AEs in a consecutive (‘real world’) patient cohort involving the most common diseases treated by DBS in the most common surgical targets. In a corresponding report the same cohort has already been evaluated for DBS treatment-inherent AEs as well as AEs related to disease progression or comorbidities [8]. Data for this cohort were contrasted with retrospective data on all DBS patients operated at our institution to address possible bias that may stem from the evaluated period. To put these institutional data into a meaningful context we performed a systematic review of 103 publications. AEs were extracted from publications and re-evaluated according to a triad of clearly defined and patient-relevant categories.

## Patients and methods

Patient demographics, indications for DBS, surgical targets, and specifics of data acquisition, grading, monitoring, evaluation and statistical analysis have been detailed in a corresponding report providing a detailed workup of AEs related to ongoing DBS therapy [8]. In addition, intra- and postoperative images of all patients were reviewed. The present analysis was performed for the purpose of internal quality control as well as proper patient counseling which should be based on actual AE rates from the treating center and not from the literature. This work is part of a doctoral thesis by one of the authors (K.E.) and was approved by the Medical Faculty of the University of Hamburg. Data entered into the database were analyzed anonymously.

Mean and median follow-up for the patients assessed (123) was 4.7 years (standard deviation 1.5 years; range 0.7 to 7.3 years). The follow-up period was <12 months for 1 patient and <24 months for 4 patients. This represents an aggregate period of 578 patient-years (4.7 years x 123 patients). We did not consider electrode-years but only patient-years as explained above.

Surgery was performed by two of the authors (WH and JAK). The procedure was performed using the same surgical technique throughout the investigated period complying with standard of care at our institution. In brief, this involved mounting a Zamorano-Dujovny stereotactic frame under general anaesthesia, MR imaging and intraoperative computed tomography (sliding gantry system; Siemens, Erlangen, Germany) followed by co-registration of the acquired images (iPlan software; Brainlab, Feldkirchen, Germany) as previously described [9]. Following extubation and placement of the burr hole, implantation of quadrupolar electrodes (model 3389; Medtronic, Minneapolis, Minnesota, USA) was guided by microelectrode recordings and microstimulation using a Ben’s gun system (AlphaOmega, Nazareth, Israel). In some instances, GPI electrode implantation and rarely STN stimulation was performed under general anaesthesia [10–12].

Intraoperative CT scanning was used to document the stereotactic electrode position and to assess for complications such as hemorrhage. The pulse generator (Kinetra, Soletra, Activa PC, or Activa RC; Medtronic, Minneapolis, Minnesota, USA**)** and extensions were implanted on the same or following day. All patients received an antimicrobial prophylaxis during surgery that was continued for an additional three days. Dexamethasone or other steroids were not administered.

AEs related to surgery or the implanted devices may vary over time depending on experience, modification of the procedure or availability of novel implants. Thus, the investigated cohort was contrasted with all patients (423) who underwent implantation of a DBS system at our institution from 2002 until December 2016. With a mean follow-up of 3.6 years this represented a cumulative follow-up of 1523 patient-years. Relevant data on AEs in our DBS patients as a whole were obtained from a complete set of surgical reports, postoperative imaging and from a prospectively acquired patient list maintained for the collection of surgery- and hardware-related complications. The last patient visit at our institution was used to determine follow-up and to calculate patient-years.

In order to compare these data with the literature a systematic review was performed. Since there is no consensus on the reporting of DBS surgery- and hardware-related AEs, an unambiguous reporting system has been created based on the following three categories: (1) intracranial AEs including hemorrhages and other intracranial complications, (2) infections, erosions and related AEs requiring partial or complete hardware removal and (3) lead revisions for various reasons including the indication for lead revision. These categories are relevant from a patient’s perspective and these offer several advantages with only few limitations as summarized in Table 1.

**Table 1:** Categories for the assessment of adverse events related to DBS surgery and implanted hardware.

Category	Items included	Patient relevance	Requirements	Advantages	Limitations
Intracranial AEs	Intracerebral hemorrhage	Risk of (1) transient or permanent neurological deficit or (2) death	Postoperative imaging	Categories cover almost all *serious* and/or *severe* DBS surgery and hardware AEs Unambiguous definition Three categories only: this does not result in the dispersal of AE rates Requiremements: selected and readily accessible source documents only Data quality is insensitive to study design and does not rely on external data monitoring; equally suited for retrospective institutional studies Useful key indicators for (1) patient counseling and (2) comparison of studies (benchmarking)	No grading of intracranial AEs Minor infections not requiring hardware removal are not covered Retrieval of cases with indications for lead revision will be dependent on the availability of clinical information
	Intraventricular hemorrhage				
	Acute subdural hematoma				
	Chronic subdural hematoma				
	Epidural hematoma				
	Subarachnoid hemorrhage				
	Brain infarction				
	Brain abscess				
	Brain edema				
Complete or partial hardware removal because of	Infection	(1) Additional surgical procedure(s) resulting in (2) interruption of DBS therapy	Complete set of surgical reports		
	Erosion				
	Ulceration				
	Wound healing abnormalities				
Lead revision or indication for lead revision because of	Lead fracture	(1) Additional intracranial procedure or (2) suboptimal outcome if revision is not performed	Complete set of surgical reports and clinical notes if lead revision has been indicated but not performed		
	Lead misplacement/malplacement				
	Lead migration				
	Lead dislocation				
	Impedance problems				
	No or suboptimal clin. effect				
	Loss of effect				

The systematic review adheres to the ‘preferred reporting items for systematic reviews and meta-analyses’ (PRISMA statement [13]; S1 Table). The ‘PubMed’ database (http://www.ncbi.nlm.nih.gov/pubmed) was searched (last search on January 7, 2017) using the following search term: ((deep brain stimulation[Title]) AND ((complication*[Title]) OR (adverse event*[Title]) OR (hemorrh*[Title]) OR (infect*[Title]) OR (hardware[Title]) OR (explant[Title]))). Additional studies were retrieved by searching the reference lists of papers by hand. The pivotal prospective and monitored DBS trials for Parkinson’s disease, tremor, or dystonia, were also included.

According to PICOS criteria the following studies were eligible: (1) *Participants* (P): Adult patients (age ≥18 years) having undergone implantation of a DBS system for movement disorders; (2) *Intervention* (I): AEs from DBS surgery or the implanted hardware within the stated follow-up period; *Comparator* (C): not applicable; control group not required; (3) *Outcomes* (O): AE rates according to three categories as defined in Table 1; (4) *Study Design* (S): all study designs except for case reports.

The retrieved articles were screened, and the following studies were excluded: case reports (32); reviews (9); articles not written in English (5); pediatric patient cohorts (2); IPG replacement studies (2; although both studies are discussed in this paper); reports presenting data that are included in later studies (2); studies with a different and non-applicable acceptation of a search term (*early motor* complications*)* (4); letter to author (1); non surgical AEs (3), e.g., about psychiatric AEs; studies related to technical defects of hardware and those dealing with MR imaging with DBS systems (2); studies about the diagnosis and management of specific AEs in selected patients cohorts, e.g. Twiddler syndrome, pneumocephalus, abscess, scalp erosion, electrode removal (9).

All studies that have remained after the first selection process were systematically reviewed (cf. flow chart). There was no additional step for the exclusion of studies based on other reasons (e.g. retrospective studies; less informative reporting).

Different approaches have been proposed for the assessement of study quality, but there is no consensus on a particular tool yet. Most tools have been tailored to the needs of intervention-related studies, and in particular recommendations for the assessment of the quality of studies evaluating surgery- and hardware-related complications are not available. Martin et al. have addressed this topic for urological surgery [14]. On the other hand, most of the proposed tools cover similar items. Hence analysis of study quality for the present systematic review will follow core questions included in most questionnaires [15–19]. As explained in Table 2, several issues are irrelevant for the present systematic review as it does not rely on results and statistics provided in the evaluated papers. In addition, a control-group and randomization are not required, and double blinded assessments are impossible for the assessment of AEs that are definitely related to DBS surgery or the implanted hardware. The most relevant issue determining study quality for the purpose of the current review has been the possibility to collect complete and accurate data from the assessed papers. On the other hand study design may have an influence on AE reporting, e.g., underreporting of AEs in studies without external data monitoring. To this end the subgroup of prospective trials involving external data monitoring was compared with other studies. To assess possible differences in AE rates that may be related to study size (number of patients) or duration (follow-up period) additional subgroup comparisons were performed.

**Table 2:** Evaluation of study quality based on questions from common check lists.

Question	Assessment and comments
Was the study question or objective clearly stated?	Objective implied by titles of papers (cf. search strategy); in addition, inclusion of prospective monitored trials (reporting of AEs is mandatory)
Was the study design appropriate for the stated aims? Was the study described as a randomized trial, a randomized clinical trial, or an RCT?	Only complete and detailed reporting of AEs is relevant for the systematic review, but not study design per se; the majority of studies were retrospective trials; a retrospective study completely and clearly reporting AEs would be more informative (higher quality) for the purpose of this review than a perfectly designed RCT presenting AEs in a summarized manner not allowing to unravel AEs according to the proposed categories (lower quality); however, data collection in a retrospective manner without independent monitoring is more likely to lead to underreporting, thus reporting of AEs from RCTs may be more complete; in addition, RCTs cover the whole spectrum of AEs; in retrospective studies actual results may have influenced the decision to publish AE data at all or actual results may have lead to the selection of data designated for publication (e.g. hemorrhages but not hardware-related AEs); RCTs were compared with studies of other design in the present systematic review
Was the study populations clearly and fully described? Were all the subjects selected or recruited from the same or similar populations? Were inclusion and exclusion criteria for being in the study prespecified and applied uniformly to all participants?	Studies include DBS patients with movement disorders; only patients implanted with DBS systems were taken into account; patients included into RCTs had been recruited according to prespecified inclusion and exclusion criteria, thus representing more selected patient cohorts
Were the cases consecutive?	With few exceptions studies stated the evaluation of consecutive cases; studies not confirming the evaluation of consecutive cases were not excluded
Were controls selected or recruited from the same or similar population that gave rise to the cases (including the same timeframe)? Were the subjects comparable? Were the groups similar at baseline on important characteristics that could affect outcomes (e.g., demographics, risk factors, co-morbid conditions)?	N/A; assessments do not require a control group, i.e. patients not having undergone DBS surgery; the AEs under investigation are definitely related to DBS surgery or the implanted hardware, and such AEs would not be observed in a non-operated control group
Was the selected period representative?	Reporting on cohorts of selected periods may lead to deviating results if the selected period is not representative for the entire length of a DBS program; studies reporting on selected periods were not excluded from this systematic review; regarding patients from our institution we have compared a cohort from a selected period with the entire group of our DBS patients
Was the length of follow-up adequate?	Follow-up was extracted from each study and cumulative follow-up in patient-years was calculated; studies not allowing such calculations are indicated; possible effects of follow-up on AE rates were investigated in the current manuscript
Was the method of randomization adequate (i.e., use of randomly generated assignment)? Was the treatment allocation concealed (so that assignments could not be predicted)?	N/A; the assessment of AEs related to DBS surgery and implanted hardware does neither require a control group nor randomization; the systematic review is based on complete and detailed reporting of AEs only
Was the intervention clearly described? Was there high adherence to the intervention protocols for each treatment group? Were other interventions avoided or similar in the groups (e.g., similar background treatments)?	N/A; with regard to the included RCTs the analysis of AEs does not take the relative effects of the studied interventions (primary endpoints) into consideration; outcome measures or other statistics presented in papers were not of interest
Were the outcome measures clearly defined, valid, reliable, and implemented consistently across all study participants? Were the results well-described?	AE rates were calculated based on primary data presented in each paper; the systematic review does not rely on statistics presented in papers; quality of data presentation was high if exact AE rates could be determined; if exact numbers could not be calculated from papers, we determined AE rates representing the lowest or highest possible rates
Were the statistical methods well-described? Did the authors report that the sample size was sufficiently large to be able to detect a difference in the main outcome between groups with at least 80% power? Was a sample size justification, power description, or variance and effect estimates provided?	N/A; the systematic review did not rely on statistics presented in papers; the number of patients implanted with DBS systems is indicated; as DBS patients are not compared with non-operated patients sample size justifications are not required for the purpose of this review; possible effects of cohort sizes on AE rates were investigated in the current manuscript
Were study participants and providers blinded to treatment group assignment? Were the people assessing the outcomes blinded to the participants’ group assignments?	N/A; blinded evaluations are impossible to perform and these are not required for the assessments of AEs that are definitely related to DBS surgery or to the implanted hardware; the assessments do not require a control group (see above)
Were all randomized participants analyzed in the group to which they were originally assigned, i.e., did they use an intention-to-treat analysis?	N/A; all patients implanted with DBS systems were taken into account but not patients from control groups; an intention-to-treat analysis is not useful for the assessment of AEs related to DBS surgery and implanted hardware
What was the overall drop-out rate from the study at endpoint?	Drop outs were taken into account for the calculation of cumulative follow-up (patient-years) if dates had been indicated by the authors

Data extraction from eligible publications involved: (1) identifying the number of patiens having received DBS implants and calculation of cumulative patient-years based on mean follow-up; (2) extraction and classification of AEs according to the proposed categories (cf. Table 1), followed by calculation of incidences based on patients and patient-years. Eligible papers were reviewed independently by two members of our group of authors and all numbers were compared. In case the numbers extracted from papers differed between two raters, papers were reassessed until consent was reached. Studies were not excluded if exact numbers could not be calculated. For example, several papers only presented the number of symptomatic hemorrhages. In this case the total number of hemorrhages was assumed to be equal or larger than the published number. Another example would be papers stating the incidence of infections but not the number of patients requiring removal of (parts of) their DBS system. In this case the number of patients with hardware removal was assumed to be less or equal to the number of reported infections.

Average AE rates, standard deviations and medians among different studies were calculated in two different ways: (i) AE rates from applicable studies were averaged, thus equal weight was given to each study irrespective of cohort size (per-study analysis); this rate indicates the average risk for a patient who would be randomly assigned to one of the treating centers that had been included into the systematic review; (ii) AE rates were also determined by pooling the incidences of a given AE among all applicable studies and by dividing this number by the total number of patients included in these studies (per-patient analysis); this rate assigns equal weight to each patient and it indicates the average risk for a patient based on all patients who had been reported on in the investigated studies.

Heterogeneity of AE rates between studies was analyzed in a random effects meta-analysis and possible effects of study size and publication year were assessed in a mixed-effects meta-regression by using the meta and the metafor packages in R version 3.4.1 [20–22]. Factors that may contribute to publication bias have been summarized in Table 3. Publication bias (cf. Table 3) was analyzed by funnel plot analysis [23] and comparisons with reported figures from large health-care related databases from the USA containing real-world data from non-academic and academic hospitals. In addition, we compared studies based on the quality of data presentation, i.e. studies for which exact rates could be calculated as opposed to studies permitting us to determine extreme (lowest or highest possible) rates only. Statistical analysis was performed with Sigmastat (Sigmastat 2.03; Systat Software Inc., Chicago, USA).

**Table 3:** Evaluation of publication bias.

Possible source of bias	Assessment and comments
Unpublished studies because of rejections based on peer reviews, journal policy or editor decisions; unsubmitted studies; uncollected AE data	Difficult or impossible to assess in a reliable manner; AE studies represent "negative" studies par exellence and may be rejected for the lack of novelty; on the other hand AE data are of interest due to their clinical relevance; actual AE rates may have an influence on peer review: high AE rates may be regarded to discredit an established procedure and low AE rates may be denied for other reasons; looking up websites of DBS centers would not reveal whether AE rates were based on own data or data from the literature and how data were collected; a survey among centers would probably result in selected responses, thus data quality and reliability would be variable; a comparison with health care-related databases appears to be the most straightforward approach and was performed for this systematic review
Selection of papers to be evaluated	The process of selecting studies for a systematic review may influence results; in the present systematic review only a single selection step for the exclusion of non-applicable studies (case reports etc.; cf. Methods) was applied
Study design	Selection of studies based on study design may influence results; as opposed to studies comparing differential effects of interventions study design per se is not relevant for the assessment of AEs definitely related to DBS surgery or hardware; in RCTs acquisition of AEs may be more complete than in retrospective studies due to data monitoring; to analyze possible underreporting monitored multi-center trials were compared with other studies; to investigate whether the quality of data presentation (cf. study quality) was associated with actual AE rates, studies were compared based on the accuracy with which AE rates could be determined
Number of subjects	Smaller studies may be less accurate and representative than larger studies; smaller studies are more likely to be drafted and accepted for publication if these report on positive findings; according to this, one may hypothesize that AE rates reported by smaller studies might be lower than those of larger studies; on the other hand, cohort size reflects clinical experience, thus smaller studies may be charged with higher AE rates; larger studies are more likely to be completed and submitted for publication due to the effort and resources spend, thus unfortunate AE rates from larger studies are more likely to be published than from smaller studies; the number of subjects included in each of the evaluated studies is indicated; possible effects of cohort sizes and follow-up on AE rates were investigated and are discussed in the manuscript
Publication date	More recent studies may report on lower AE rates because of technical advances and higher medical standards; on the other hand, the date of publication is delayed by the length of follow-up; longer follow-up also increases the chance to collect hardware-related AEs; in addition, the initial treatment of a patient with long follow-up may have been according to outdated medical standards; a possible effect of publication date was investigated

## Results

Among a total of 433 AEs, 23 AEs (5.3%) were related to surgery or the implanted hardware and affected 18 patients (14.6% of 123 patients). All AEs related to the surgical procedure or implanted hardware are presented in detail in S2 Table. The age of patients affected or non-affected by surgery- and hardware-related AEs did not differ (p> 0.05; Mann-Whitney U Test).

### Intracranial complications

**Fig 1:**
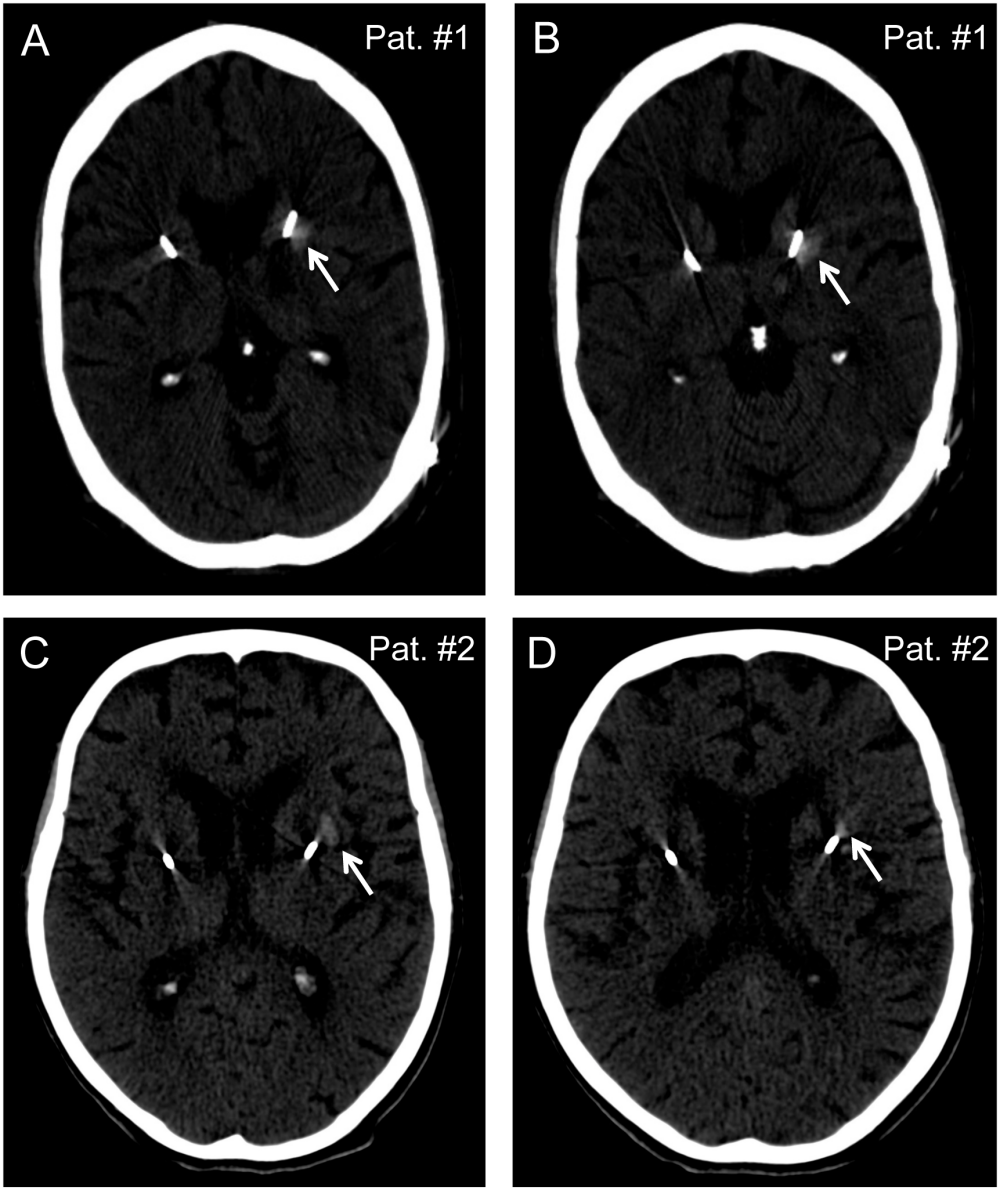
Intracerebral hemorrhages in the striatum along implanted electrodes (arrows) as detected in CT scans obtained on postoperative day three (A and B, patient#1; STN stimulation; diameter 1.2 cm) and day one (C and D, patient#2; GPI stimulation; diameter < 1 cm).

In two cases, a small hemorrhage in the striatum along one of the implanted electrodes was detected (Fig 1 and S2 Table). The maximum extent of these hemorrhages was observed in repeated CT scans obtained on postoperative day three (patient#1; diameter 1.2 cm) or day one (patient#2; diameter < 1 cm; Fig 1).

Both hemorrhages went along with transient impairments that resolved completely. A 65-year-old male suffering from Parkinson’s disease developed moderate deficits in cognition and word fluency. These had improved significantly until discharge and had resolved completely within the following three months. A 75-year-old female suffering from segmental dystonia revealed postoperative dysphasia, gait disturbances with delayed mobilization and problems with movement initiation (but normal strength after full innervation) contralateral to the hematoma. Symptoms improved during the hospital stay and fully resolved within three months. Both patients were suffering from hypertension and coronary artery disease involving prior (in one case repeated) coronary artery stenting. Both patients had discontinued aspirin one week prior to surgery.

The rate of intracranial complications in this cohort (1.6%; n = 123 patients) is slightly lower than among all patients implanted with DBS electrodes at our institution until 2016 (2.1%; n = 423).

### Hardware removal and other infection-related AEs without hardware removal

In four patients (3.3%) IPG removal was required because of infection (three patients were reimplanted). The pertinent information is summarized in S2 Table. In only one case, a 60-year-old patient suffering from insulin-dependent diabetes mellitus, infection (Staphylococcus aureus) occurred within the first 3 months. One 69-year-old patient suffered from repeated infections at (different) IPG sites that required an additional course of explantation and replantation. The ages of the other affected patients were 70 (non-purulent chronic inflammation with erosion) and 64 years (purulent infection).

Other wound complications or circumscribed secondary wound healing (four patients; 3.3%) were treated by local revisions (S2 Table). Until preparation of this manuscript these four patients have had between 67 and 119 months follow-up without the recurrence of infections or erosions in the re-operated areas. The ages in these patients (17 to 75 years) had a broader range.

All AEs involving hardware infections were reversible. In the present cohort (and all other patients implanted with DBS systems at our center) we never observed infections at the burr hole or intracranially.

The rates for hardware removal in this cohort of 123 patients (3.3% and 0.7% per patient-year) were similar to all patients implanted with DBS electrodes at our institution until 2016 (n = 423**;** 2.6% and 0.7% per patient-year).

### Lead revisions and other device-related AEs

One defective electrode (low impedance for several contacts) was replaced one week after implantation (S2 Table). There was no lead migration or surgical revision because of lead misplacement. The rates for electrode revisions in this cohort of 123 patients (1.6% and 0.3% per patient-year) were similar to all patients implanted with DBS electrodes at our institution until 2016 (n = 423; 1.4% and 0.4% per patient-year).

### Systematic review of literature

For a meaningful comparison of the present cohort with other studies we systematically reviewed 103 studies. Eligible studies were identified as described above and this process is shown in the flow diagram (S1 Fig). In many instances exact numbers could not be derived (Table 4). This was due to missing information. Typical examples for this are: (1) the reporting of symptomatic hemorrhages only; (2) the reporting of a number of SAEs without specifying these; (3) the reporting of infections without giving further details about consequences (e.g. explantation of hardware); (4) the reporting of surgical revisions without indicating electrode revisions.

**Table 4:** Systematic review of the literature based on a triad of categories.

Author	Pat.	F/U	Pt.yrs	Intracranial AEs	Hardware removal		Lead revision	
	n =	mean		n (%)	n (%)	% per pt.yrs	n (%)	% per pt.yrs
**White-Dzuro, 2016** [24]	291	2.8	815	ND	< >9 (< >3.1)	< >3.1	12 (4.1)	1.5
**Rasouli, 2016** [25]	179	ND	ND	ND	1 (0.6)	ND	ND	ND
**Petraglia, 2016** [26]	713	0.25	178	20 (2.8)	35 (<4.9)	<19.7	38 (5.3)	21.3
**Rosa, 2016** [27]	105	1	105	ND	3 (2.9)	2.9	ND	ND
**Wang, 2016** [28]	396	ND	ND	≥10 (≥2.5)	ND	ND	ND	ND
**Levi, 2015** [29]	107	0.25	26.8	5 (4.7)	1 (0.9)	3.7	0 (0)	0
**Falowski, 2015** [30]	432	>>0.5	>>216	24 (5.6)	10 (2.3)	<<4.6	34 (7.9)	<<15.7
**Verla, 2015** [31]	661	0.25	165	≥10 (≥1.5)	≤20 (≤3)	≤12.1	2 (0.3)	1.2
**Patel, 2015** [32]	392	4.1	1611	24 (6.1)	≤19 (≤4.8)	≤1.2	>20 (>5.1)	>1.2
**Timmermann, 2015** [33]	40	1	40	0 (0)	≤1 (≤2.5)	≤2.5	0 (0)	0
**Gocmen, 2014** [34]	102	ND	ND	ND	6 (5.9)	ND	ND	ND
**DeLong, 2014** [35]	1757	0.25	439.3	≥25 (≥1.4)	≤64 (≤3.6)	≤14.6	30 (1.7)	6.8
**Zibetti, 2014** [36]	221	ND	ND	0 (0)	ND	ND	ND	ND
**Bjerkness, 2014** [37]	368	1	368	ND	26 (7.1)	7.1	ND	ND
**Tolleson, 2014** [38]	447	>1	>447	ND	≤26 (≤5.8)	<5.8	ND	ND
**Volkmann, 2014** [39]	62	0.5 /0.75	38.5	1 (1.6)	≥2 (≥3.2)	≥5.2	≥3 (≥4.8)	≥7.8
**Seijo, 2014** [40]	233	7.1	1654	10 (4.3)	ND	ND	26 (11.2)	1.6
**Fenoy, 2014** [41]	728	>>1.9	>>1383	40 (5.5)	13 (1.8)	<<0.9	41 (5.6)	<<3.0
**Piacentino, 2013** [42]	106	ND	ND	5 (4.7)	ND	ND	ND	ND
**Pepper, 2013** [43]	273	ND	ND	ND	11 (4)	ND	ND	ND
**Schuepbach, 2013** [44]	120	2	240	*26 SAE ‘related to surgery or device’ of which 11 (42%) were not specified; 1 intracerebral abscess; 1 edema*				
**Oderkerken, 2013** [45]	128	1	<128	3 (2.3)	≤4 (≤3.1)	< >3.1	0 (0)	0
Rughani, 2013* [46]	4961	ND	ND	< >90 (< >1.8)	ND	ND	ND	ND
**Halpern, 2012** [47]	165	1.9	325	ND	10 (6.1)	3.1	ND	ND
**Dlouhy, 2012** [48]	100	ND	ND	ND	9 (9)	ND	ND	ND
**Guridi, 2012** [49]	110	ND	ND	ND	ND	ND	5 (4.5)	ND
**Volkmann, 2012** [6]	32	>5	>160	ND	≤7 (≤21.9)	<4.4	≥9 (≥28)	< >5.6
**Kahn, 2012** [50]	15	>0.25	too low	1 (6.7)	0 (0)	ND	0 (0)	ND
**Okun, 2012** [51]	136	1	136	4 (2.9)	≤6 (≤4.4)	≤4.4	3 (2.2)	2.2
**Baizabal C., 2012** [52]	512	3.9	1997	ND	10 (1.9)	0.5	32 (6.3)	1.6
**Falowski, 2012** [53]	326	>> .5	>>163	16 (4.9)	9 (2.8)	<<5.5	14 (4.3)	<<8.6
**Zrinzo, 2012** [54]	214	ND	ND	2 (0.9)	ND	ND	ND	ND
**Park, 2011** [55]	110	ND	ND	9 (8.2)	ND	ND	ND	ND
**Piacentino, 2011** [56]	106	1	106	ND	9 (8.5)	8.5	ND	ND
**Fily, 2011** [57]	67	ND	ND	ND	≤6 (≤8.9)	ND	ND	ND
**Servello, 2011** [58]	272	ND	ND	ND	7 (2.6)	ND	2 (0.7)	ND
**Doshi, 2011** [59]	153	5.3	816	2 (1.3)	5 (3.3)	0.6	4 (2.6)	0.5
**Foltynie, 2011** [60]	79	1.2	92.4	1 (1.3)	0 (0)	(0)	1 (1.3)	1.1
**Fisher, 2010** [61]	110	3	330	5 (4.5)	9 (8.2)	2.7	9 (8.2)	2.7
**Fytagoridis, 2010** [62]	40	2.8	113.3	0 (0)	1 (2.5)	0.9	1 (2.5)	0.9
**Bhatia, 2010** [63]	270	5.5	1485	ND	20 (7.4)	1.3	ND	ND
**Williams, 2010** [3]	178	1	178	3 (1.7)	≤16 (≤9)	≤9	≥0 (≥0)	≥0
**Boviatsis, 2010** [64]	106	2.5	265	2 (1.9)	4 (3.8)	1.5	2 (1.9)	0.8
**Fernandez, 2010** [65]	208	4.25	884	ND	≤6 (≤2.9)	≤0.7	14 (6.7)	1.6
**Zhang, 2010** [66]	34	4.7	161.2	ND	3 (8.8)	1.9	4 (11.8)	2.5
**Follett, 2010** [67]	299	<2	<598	9 (3)	≤23 (≤7.7)	< >3.8	≤6 (≤2.0)	< >1.0
**Vergani, 2010** [68]	141	4.6	649	2 (1.4)	6 (4.3)	0.9	2 (1.4)	0.3
**Burdick, 2010** [69]	198	0.5	99	≥29 (≥14.6)	7 (3.5)	7.1	17 (8.6)	17.2
**Xiaowu, 2010** [70]	137	ND	ND	1 (0.5)	ND	ND	ND	ND
**Sixel-Döring, 2010** [71]	85	<3	>211	ND	≥8 (≥9.4)	< >3.8	ND	ND
**Hu, 2010** [72]	161	1.2	193	1 (0.6)	2 (1.2)	1	2 (1.2)	1
**Okun, 2009** [73]	52	>0.6	>31.2	6 (11.5)	≥2 (≥3.8)	< > 6.4	ND	ND
**Elias, 2009** [74]	143	ND	ND	20 (14)	ND	ND	ND	ND
**Maldonado, 2009** [75]	194	4.1	795.4	0 (0)	2 (1.0)	0.3	9 (4.6)	1.1
**Ben-Haim, 2009** [76]	130	ND	ND	7 (5.4)	ND	ND	ND	ND
**Gervais-Bern., 2009** [77]	42	5	>115	2 (4.8)	4 (9.5)	<3.5	2 (4.8)	<1.7
**Chan, 2009** [78]	55	ND	ND	≥1 (≥1.8)	1 (1.8)	ND	5 (9.1)	ND
**Weaver, 2009** [2]	121	<0.5	<60.5	≥1 (≥0.8)	12 (9.9)	19.8	≤8 (≤6.6)	≤13.2
**Hariz, 2008** [79]	69	4	276	ND	≥2 (≥2.9)	≥0.7	≥1 (≥1.5)	≥0.4
**Khatib, 2008** [80]	250	ND	ND	7 (2.8)	ND	ND	ND	ND
**Wider, 2008** [81]	50	<5	>185	>0 (>0)	1.0 (2.0)	<1.1	3 (6.0)	<1.6
**Schuurman, 2008** [82]	31/25	2/5	137	ND	1 (2.9; n = 34)	0.7	1 (2.9; n = 34)	0.7
**Bhatia, 2008** [83]	191	4.9	931	7 (3.7)	14 (7.3)	1.5	<14 (<7.3)	<1.5
**Sillay, 2008** [84]	420	4.3	1806	ND	20 (4.8)	1.1	ND	ND
**Derost, 2007** [85]	87	1.3	117	≥0 (≥0)	≤1 (≤1.1)	≤0.9	ND	ND
**Seijo, 2007** [86]	130	3.1	400.8	9 (6.9)	2 (1.5)	0.5	6 (4.6)	1.5
**Chou, 2007** [87]	26	0.7	18.2	1 (3.8)	0 (0)	0	≥2 (≥7.7)	≥11
**Vidailhet, 2007** [88]	22	3	66	ND	1.0 (4.5)	1.5	2.0 (9.0)	3.0
**Sansur, 2007** [89]	115	ND	ND	≥4 (≥3.5)	ND	ND	ND	ND
**Ory-Magne, 2007** [90]	45	<2	<90	≥4 (≥8.9)	≤5 (≤1.1)	<5.6	ND	ND
**Vesper, 2007** [91]	73	<2	<116	≥1 (≥1.4)	5 (6.9)	>4.3	ND	ND
**Kenney, 2007** [92]	319	2.8	893	5 (1.6)	≤8 (≤2.5)	≤0.9	17 (5.3)	1.9
**Tir, 2007** [93]	100	1	100	6 (6.0)	4 (4.0)	4.0	12 (12.0)	12.0
Voges, 2007** [94]	<<1183	0.08	<<98.6	>26 (>2.2)	5 (0.4)	>5.1	ND	ND
**Voges, 2006** [95]	262/180	0.08/3	545	≥1 (≥0.4)	12 (6.7)	2.2	8 (4.4)	1.5
**Paluzzi, 2006** [96]	60	3.6	216	ND	≤7.0 (≤11.7)	≤3.2	≤17.0 (≤28.3)	≤7.9
**Goodman, 2006** [97]	100	>0.5	>50	3 (3.0)	5 (5.0)	<10.0	5 (5.0)	<10.0
**Kupsch, 2006** [98]	40	0.5/0.75	>25	0 (0)	2 (5.0)	8.0	2 (5.0)	8.0
**Blomstedt, 2006** [99]	129	3.9	503	1 (0.8)	<7 (<5.4)	<1.0	10 (7.8)	2.0
**Deuschl, 2006** [1]	76	0.5	38	3 (3.9)	≤2 (≤2.6)	≤5.3	≥0 (≥0)	≥0
**Vidailhet, 2005** [100]	22	1	22	1 (4.5)	0 (0)	0	1 (4.5)	4.5
**Binder, 2005** [101]	280	ND	ND	16 (5.7)	ND	ND	ND	ND
**Constantoy., 2005** [102]	144	2	288	ND	8 (5.6)	2.8	0 (0)	0
**Visser-Vand., 2005** [103]	32	4.5	90	0 (0)	0 (0)	0	0 (0)	0
**Schuepbach, 2005** [104]	37	<5	>157	0 (0)	1 (2.7)	<0.6	3 (8.1)	<1.9
**Blomstedt, 2005** [105]	119	>1	<393	ND	≥2 (≥1.7)	>0.5	10 (8.4)	2.5
**Coubes, 2004** [106]	31	3.5	108.5	0 (0)	1 (3.2)	0.9	≥0 (≥0)	≥0
**Temel, 2004** [107]	106	3.6	367.7	ND	2 (1.9)	0.5	ND	ND
**Lyons, 2004** [108]	81	ND	ND	1.0 (1.2)	5 (6.2)	ND	17 (21.0)	ND
**Krack, 2003** [109]	49	5	<245	10 (20.4)	1 (2.0)	>0.4	0 (0)	0
**Umemura, 2003** [110]	109	1.7	182	≥5 (≥4.6)	4 (3.7)	2.2	ND	ND
**Herzog, 2003** [111]	48	1.3	60	2 (4.2)	0 (0)	0	0 (0)	0
**Pollak, 2002** [112]	300	ND	ND	15 (5.0)	10 (3.3)	ND	7 (2.3)	ND
**Starr, 2002** [113]	44 (45)	<1	<44	3 (6.7)	0 (0)	0	2 (4.5)	>4.5
**Oh, 2002** [114]	84	2.8	217	3 (3.6)	12 (14.3)	5.5	10 (11.9)	4.6
**Kondziolka, 2002** [115]	66	2.4	159.5	1 (1.5)	9 (13.6)	5.6	13 (19.7)	8.2
**Joint, 2002** [116]	39	1	39	ND	0 (0)	0	6 (1.5)	1.5
**Vesper, 2002** [117]	129	0.4	52	4 (3.1)	≤7 (≤5.4)	≤13.5	≥2 (≥1.6)	≥3.8
**Beric, 2001** [118]	86	ND	ND	3 (3.5)	0 (0)	ND	3 (3.5)	ND
**PD study, 2001** [119]	134	<0.5	<70	7 (5.2)	2 (1.5)	>2.9	9 (6.7)	>12.9
**Koller, 2001** [120]	49	>2	>98	6 (12.2)	1 (2.0)	<1.0	10 (20.4)	<10.2
**Schuurman, 2000** [5]	34	0.5	17	1 (2.9)	1 (2.9)	5.9	0 (0)	0
**Benabid, 2000** [121]	127	ND	ND	8 (6.3)	ND	ND	ND	ND
**UKE, present study**	123	4.7	578	2 (1.6)	4 (3.3)	0.7	2 (1.6)	0.3
**UKE, 2002–2016**	423	3.6	1523	9 (2.1)	11 (2.6)	0.7	6 (1.4)	0.4

Systematic review of DBS studies reporting AEs since the year 2000, including monitored prospective and randomized trials. The number of AEs was calculated based on patients and not procedures. Patient numbers refer to patients who actually underwent surgery. If the exact incidence of AEs or cumulative follow-up could not be determined, this was indicated by the use of “≥” or “≤“. Intracranial AEs include intracranial hematomas, brain abscesses, brain infarction, and brain edema. In several instances only symptomatic hematomas were reported, which was indicated with ‘≥’. Explantation because of infection or erosion includes both partial and complete hardware removal. Patients affected by multiple infections or procedures were only counted once. Electrode AEs include lead revisions because of migration, fracture, misplacement or replacement of leads due to loss of effect. ND, not assessed or presented by authors, or data could not be rated in a reliable manner.

*mostly unilateral procedures and symptomatic hemorrhages were probably captured only; risk of hemorrhage was 2.7% for bilateral procedures (n = 450);

**only number of procedures stated (patient number not deducible)

**Fig 2:**
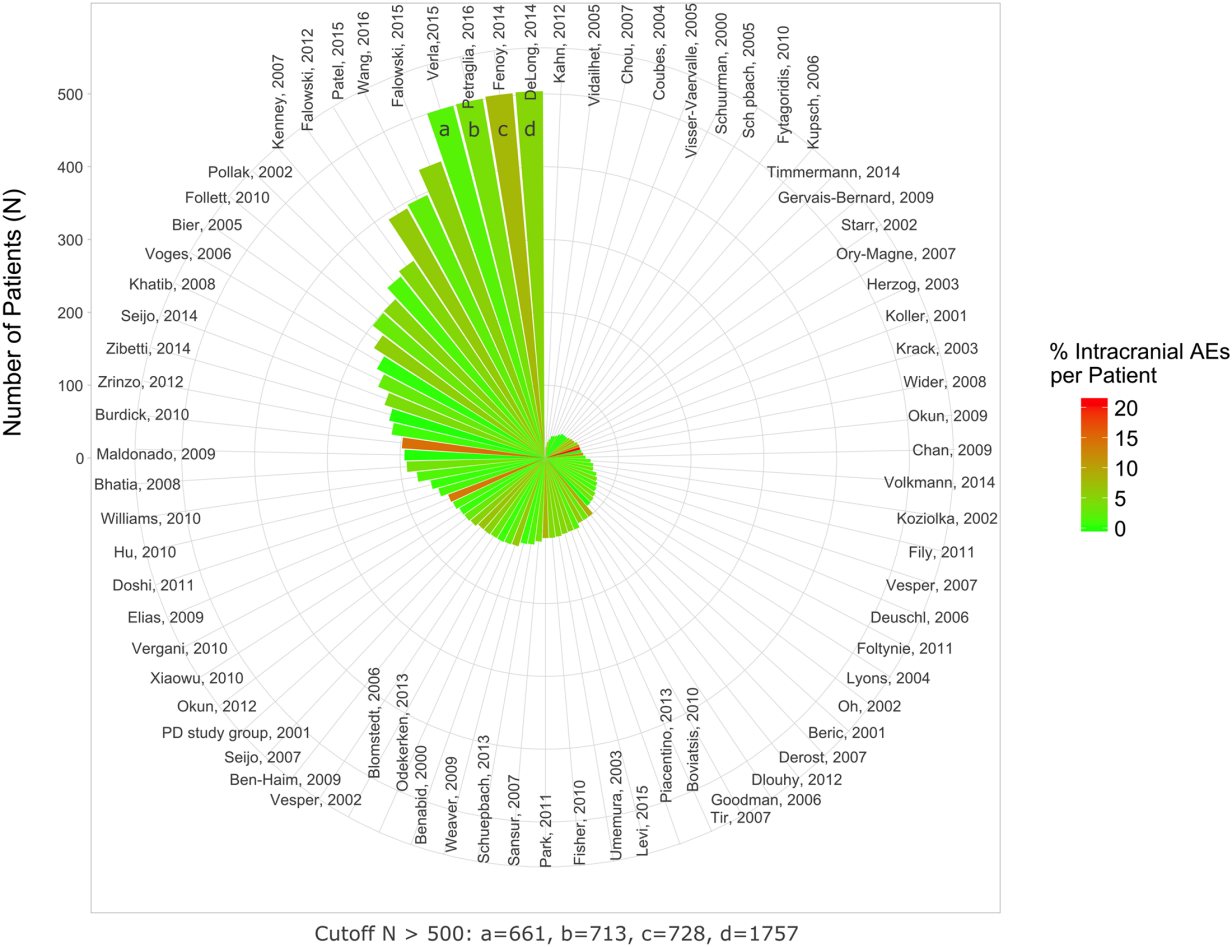
Percent intracranial AEs per patient. The rate of AEs is color-coded. The number of patients that have been included in respective studies are indicated by the height of the wedges (cutoff value 500 patients).

The average rate of intracranial complications was 3.8% and 3.4% for per-study and per-patient analysis, respectively (Table 5 and Fig 2). For intracranial complications there was a significant degree of heterogeneity between studies (random effects logistic meta-analysis; I^2^ = 77.4%; p< 0.0001; S2 Fig). In a mixed-effects meta-regression the degree of heterogeneity was not reduced significantly by taking the number of patients included in respective studies (p> 0.05; S3 Fig) or publication year (p> 0.05) into account (residual heterogeneity I^2^ = 76.2%; p< 0.001). Significant funnel plot asymmetry was found (p< 0.001, linear regression test of funnel plot asymmetry; S4 Fig).

**Table 5:** Summary of literature-based rates for adverse events related to DBS surgery and implanted hardware.

	Applicable studies	Incidence		Incidence per-patient-year	
		per-study	per-pat.	per-study	per-pat.
**Intracranial AEs**	n = 75	3.8 (3.8; 3.0)	3.4		
**Hardware removal**	n = 86 & 77	4.3 (3.7; 3.2)	3.8	3.6 (4.2; 2.2)	2.4
**Lead revision**	n = 69 & 63	5.8 (6.2; 4.6)	4.5	4.1 (5.0; 1.6)	2.6

Summary of adverse events related to DBS surgery and implanted hardware as analyzed from studies published between 2000 and 2016. The categories ‘intracranial AE’, ‘hardware removal’ and ‘lead revision’ are explained in Table 1. The numbers represent mean (standard deviation; median) percentages as calculated from Table 4. The number (n =) of applicable studies is indicated. For hardware removal and lead revision two numbers are presented (e.g. n = 38 & 34) indicating the number of applicable studies for the analysis of ‘Incidence’ (n = 38) and ‘Incidence per-patient-year’ (n = 34). Per-study, mean values represent the average of percentages that have been calculated among eligible studies (i.e., the same relative weight is given of each study irrespective cohort size). Per pat., the total number of patients affected by respective AEs in applicable studies was summed up and divided by the total number of patients included in these studies. For calculation of ‘intracranial AEs’ the studies by Rughani, 2013 and Voges, 2007 were excluded as the actual number of individual patients could not be determined (cf. Table 4).

**Fig 3:**
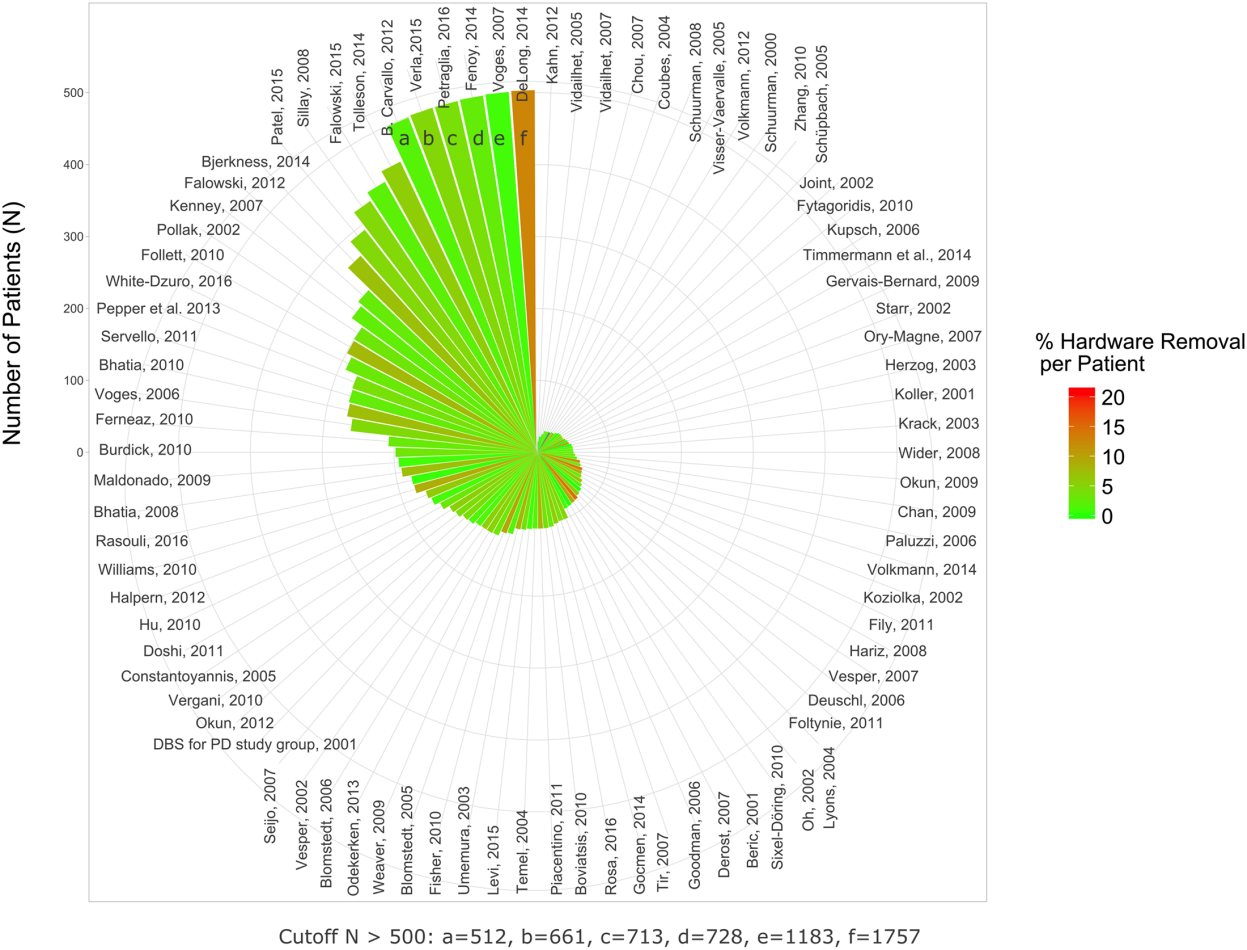
Percent hardware removal per patient. The rate of AEs is color-coded. The number of patients that have been included in respective studies are indicated by the height of the wedges (cutoff value 500 patients).

**Fig 4:**
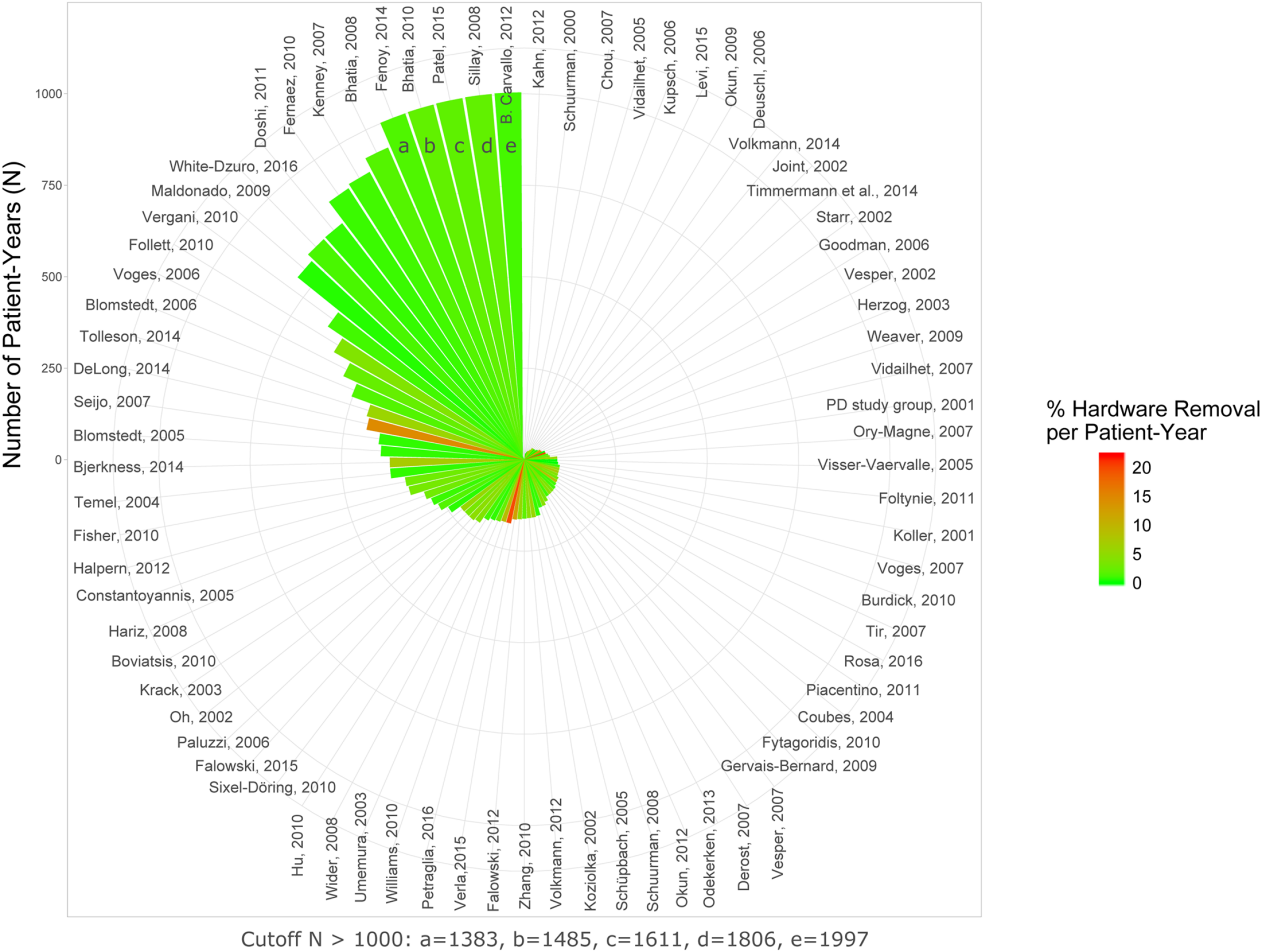
Percent hardware removal per patient-year. The rate of AEs is color-coded. The number of patient-years calculated for individual studies is indicated by the height of the wedges (cutoff value 1000 patient-years).

The rates for hardware removal were 4.3% and 3.8% for per-study analysis and per-patient analysis, respectively (Table 5; Figs 3 and 4). Similarly, evaluations based on patient-years (i.e., the reported follow-up is taken into account) revealed lower annual rates for per-patient analysis than for per-study analysis (Table 5). Lower rates obtained for per-patient analysis suggest that hardware removal rates may be lower in larger studies. For hardware removal there was a significant degree of heterogeneity between studies (random effects logistic meta-analysis based on per patient-year rates; I^2^ = 87.4%; p< 0.0001; S5 Fig). In a mixed-effects meta-regression the degree of heterogeneitiy was slightly reduced by taking study size (p< 0.0001; S6 Fig) and publication year (p = 0.006) into account, although a significant amount of residual heterogeneity remained (I^2^ = 80.0%; p< 0.0001). The meta-regression indicated that rates were lower in studies with higher cumulative patient-years or published more recently (estimates of –0.32 and 0.09, respectively). Significant funnel plot asymmetry (p< 0.001) was detected (S7 Fig).

**Fig 5:**
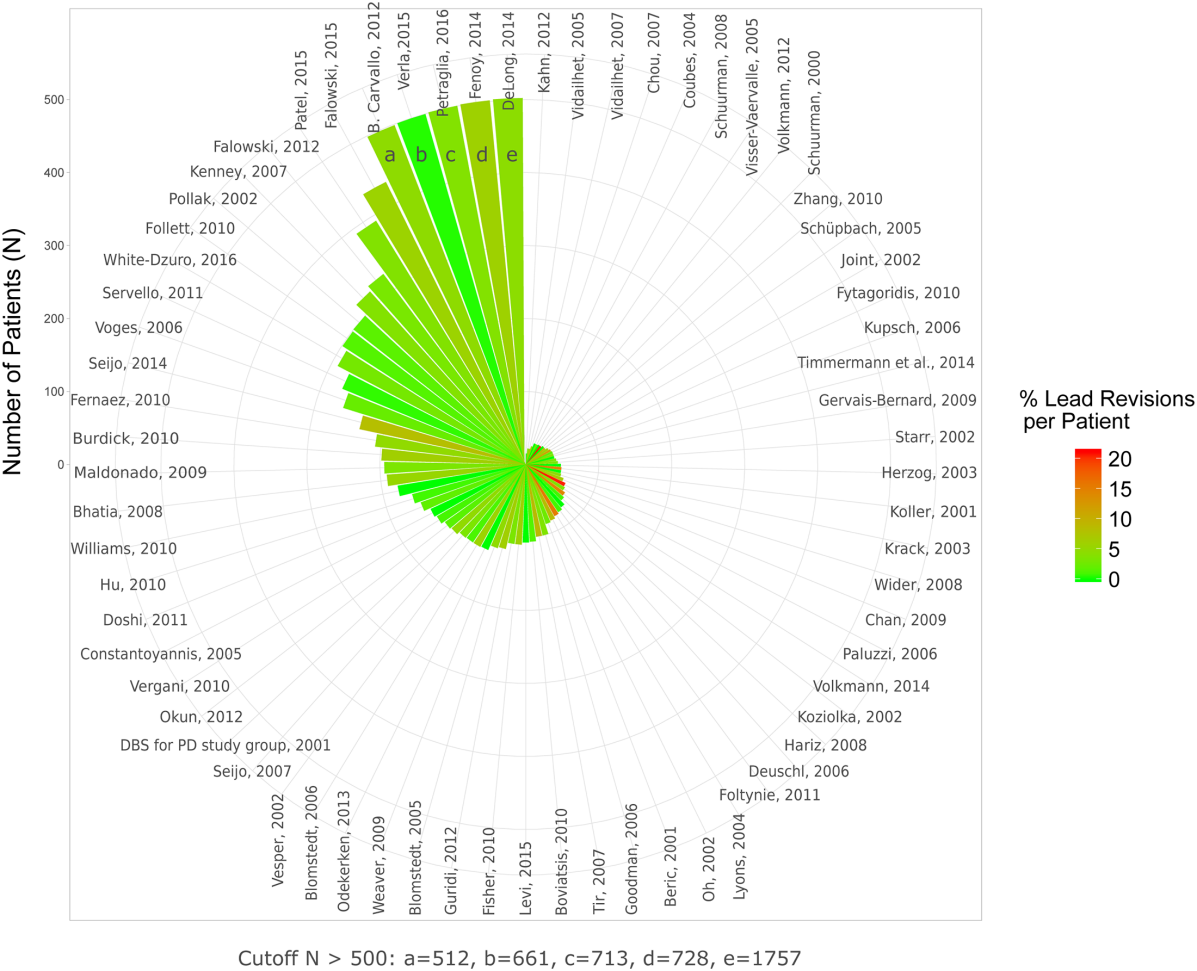
Percent lead revision per patient. The rate of AEs is color-coded. The number of patients that have been included in respective studies are indicated by the height of the wedges (cutoff value 500 patients).

**Fig 6:**
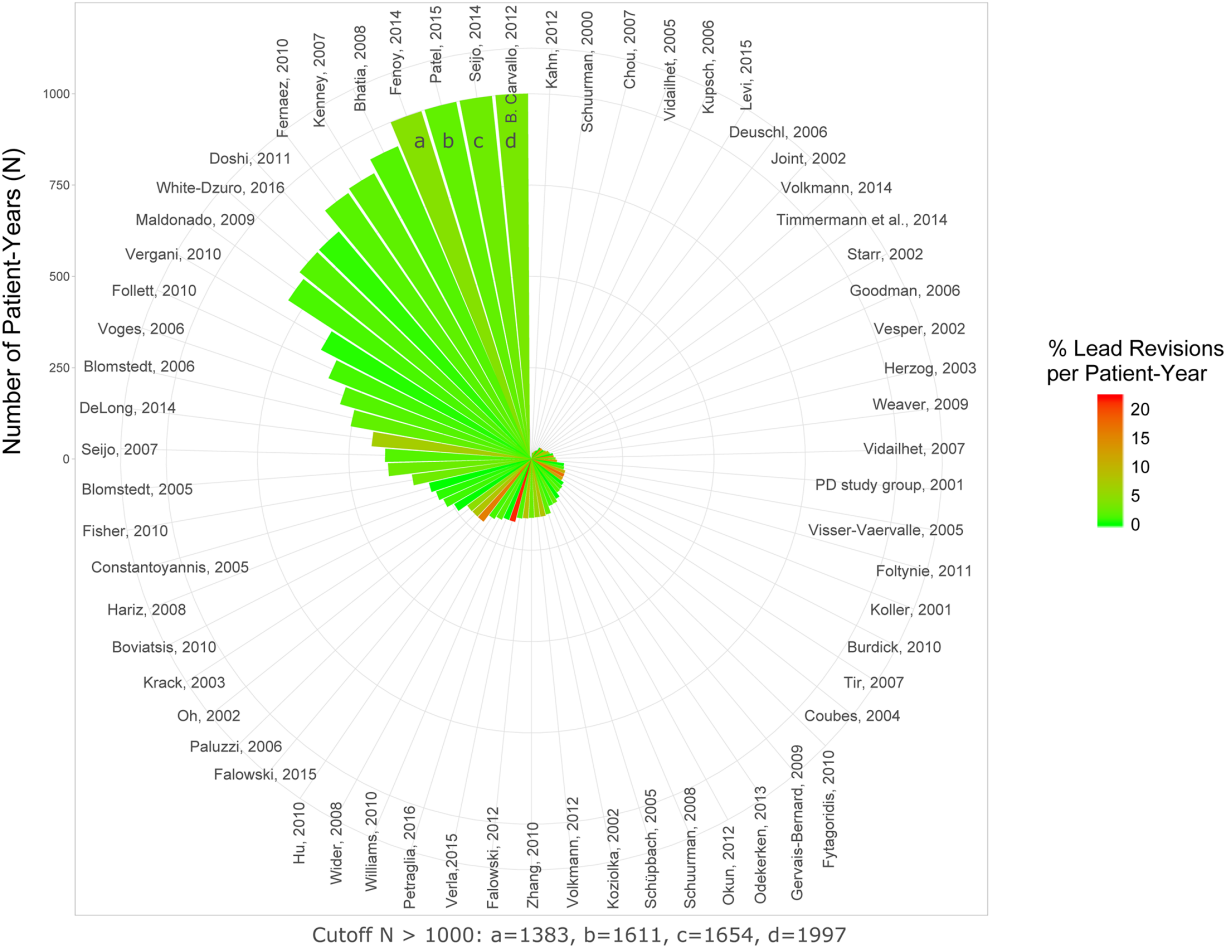
Percent lead revision per patient-year. The rate of AEs is color-coded. The number of patient-years calculated for individual studies is indicated by the height of the wedges (cutoff value 1000 patient-years).

For lead revisions the incidences were 5.8% and 4.5% for per-study and per-patient analyis, respectively (Table 5; Figs 5 and 6). Similar to hardware removal, this suggested that rates might be lower in larger studies. For lead revisions there was a significant degree of heterogeneity between studies (random effects logistic meta-analysis based on per patient-year rates; I^2^ = 91.2%; p< 0.0001; S8 Fig). In a mixed-effects meta-regression the degree of heterogeneity was slightly reduced by taking study size (p< 0.02; S9 Fig) but not publication year; p> 0.5) into account, although the degree of remaining heterogeneity was significant (I^2^ = 88.6%; p< 0.0001). A negative meta-regression estimate of –0.23 indicated that rates were lower in studies with higher cumulative patient-years. Significant funnel plot asymmetry (p< 0.015) was observed (S10 Fig).

To investigate whether AE rates were associated with the quality of data presentation we compared studies from which exact AE rates could be determined with studies permitting to calculate extreme (lowest or highest possible) rates only (cf. Table 4). For hardware removal and lead revision average rates were lower in studies from which exact rates could be determined (Table 6). Although none of the differences were of statistical significance (p> 0.05; ANOVA), a systematic review that had been limited to studies with exactly calculable rates had resulted in lower mean rates for hardware removal and lead revision.

**Table 6:** AE rates dependent on quality of AE reporting.

	Per-patient analysis				Per-patient-year analysis			
	Exact calculation of AE rates was possible	True AE rates are			Exact calculation of AE rates was possible	True AE rates are		
		≤ or < than calculated rates	≥ or > than calculated rates	< or > than calculated rates		≤ or < than calculated rates	≥ or > than calculated rates	< or > than calculated rates
**Intracranial AEs**	4.0 (3.7)		3.2 (4.0)					
**Hardware removal**	3.8 (3.3)	5.6 (4.7)	4.2 (3.0)	*3*.*1*	2.7 (3.7)	5.3 (5.2)	2.7 (2.2)	4.3 (1.7)
**Lead revision**	5.1 (5.0)	9.1 (9.6)	7.0 (9.6)		2.9 (4.6)	6.1 (5.1)	5.2 (4.9)	*1 and 5*.*6*

Mean percentages (standard deviation) are indicated. Values are based on at least 5 studies with two exceptions for which single rates for the according studies were presented (in italics). None of the differences proved to be statistically significant (p > 0.05; ANOVA).

## Discussion

This study provides comprehensive short- and long-term data on complications related to DBS surgery or the implanted devices. In the present cohort all AEs related to surgery and the implanted hardware were reversible and resolved without permanent sequelae. To obtain comparable data about the frequency of such AEs in the literature a systematic review was performed on 103 studies involving a reassessment of AE rates based on a triad of clearly defined categories.

### Intracranial complications

The incidence of intracranial AEs at our institution compares favorable with rates determined from a systematic literature review. It is disputed whether age and hypertension represent true risk factors for intracranial hemorrhages as, for example, age may only serve as a surrogate for medical comorbidity (e.g. [54, 94, 122]). In our cohort, hemorrhages occurred at the ages of 65 and 75, and both patients were suffering from hypertension and coronary artery disease treated with coronary artery stenting. Both patients had discontinued aspirin one week prior to surgery. As a consequence from these two cases, we now require patients to discontinue aspirin for two weeks prior to surgery.

Microelectrode recordings (MER) have been related to an increased risk of intracerebral hemorrhages [54, 89, 101, 123]. Nevertheless, it has been demonstrated that MER can also be performed with a very low risk of hemorrhagic complications (e.g. [36]). In consideration of the potential risks, we limit the number of microelectrode passes to a minimum, but continue to regard MER very highly as these generate patterns that provide a high level of confidence for proper electrode implantation. Since 2002 the position of only one electrode has been revised. In this case a properly placed GPI lead was repositioned within the GPI 14 months after primary implantation, which, however, did not result in increased efficacy. In another patient we recommended revision of a suboptimally placed VIM electrode, but surgery was declined. We attribute our low rates of lead revisions to the information gathered from MER.

### Hardware removal

Hardware removal rates in the investigated cohorts appear to be relatively low when compared to the literature. This might be due to the fact that a tried and tested implantation procedure has been adopted from the beginning. In 6 of 11 (2.6%) cases non-purulent chronic inflammation had resulted in erosion of the skin and occured late. This number also includes three non-purulent infections that occurred between 17 and 32 months after IPG replacement (see below). In rare instances, chronic inflammation might be due to an abnormal foreign body reaction [124–126], but it is unclear whether this played a role in the patients affected. It would be desirable to obtain a histological workup in future cases. One patient suffered from mechanical erosion at the IPG site caused by backpacking.

In contrast to other reports [37, 43, 127–129], we found that the risk for device infection after IPG replacements is lower than after primary implantation. We have performed >330 IPG replacement procedures since 2002 without purulent infections. In three patients there were long-term complications in the form of chronic inflammation or erosion that had occurred between 17 and 32 months after IPG replacement.

### Lead revisions

Lead revison rates in the investigated cohorts were lower than those from a systematic literature review. One patient required a lead replacement after low impedances were detected for one of the electrodes during monopolar review. Since we could not rule out that the miniplate used for lead fixation had caused a short circuit, we now use a piece of silicon to pad the miniplate. This problem has not been observed in the cases operated thereafter. We did not observe any electrode dislocations. It is now common knowledge that the connector should not be placed into the neck region as this had represented one of the most crucial risk factors for lead dislocation or fracture [130, 131].

### Systematic review of literature

When we attempted to relate the surgery- and hardware-related AEs of our cohort with the literature we realized fundamental, yet largely unrecognized problems related to inconsistent reporting, which required the recalculation of AE rates according to unambiguous categories (cf. Table 1). In their entirety the three proposed categories cover most surgery- and hardware-related complications that would require a rating as *severe* and *serious*. Nonetheless, exact numbers could not be derived from many publications including monitored prospective trials. Thus it had not been possible to collect more precise data by restricting the present systematic review to monitored prospective trials only. On the other hand, the exclusion of less informative studies had resulted in lower mean AE rates (cf. Table 6). Hence it represented a more conservative approach to refrain from a second selection step elimating such studies.

A significant degree of heterogeneity between studies was observed for all three categories. With regard to hardware removal and lead revision, by meta-regression analysis a significant, but only minor degree of heterogeneity was explained by study size, with lower rates in studies with higher cumulative patient-years. Funnel plot asymmetry was found for all three categories and may be explained by publication bias with a relative lack of studies reporting higher rates of AEs. This gains support from recent analyses of health care-related databases reporting rates for lead revision and hardware removal that were markedly higher than the figures derived from the present systematic literature review [26, 31, 35, 132].

### Strengths and limitations of the study

A consecutive (’real world’) patient cohort was investigated reflecting the most common diseases treated by DBS and the most common surgical targets. This study has the advantage of exclusion from the unavoidable selection bias of prospective studies that recruit patients according to defined inclusion criteria. Thus, our cohort is likely to represent patient populations similar to those of other DBS centers. Follow-up was relatively homogeneous and comparably long (cf. Table 4). Complication rates did not decline over time, which may be explained by the fact that a tried and tested procedure has been used from the beginning.

Possible limitations of the present study might be its retrospective design and lack of independent data monitoring. Prospective reporting has been demonstrated to lead to higher AE rates [69]. However, precisely for that reason we have created categories that are based on AEs involving readily accessible and archived source documents (i.e., postoperative imaging, surgical reports), which minimizes the risk of underreporting.

## Conclusions

The incidence of adverse events related to surgery or DBS implants in the present cohorts compare favorable with rates derived from a systematic review of the literature. Benchmarking was based on three categories that are clearly defined and relevant from a patient’s perspective. The categories require no more than selected and readily accessible source documents, and they are applicable to retrospective assessments. Although the categories cover mostly severe and serious AEs, a systematic re-evaluation of 103 publications revealed that exact AE rates could not be determined from many studies. This could not be attributed to study designs. There was significant heterogenity between studies for all three categories. For hardware removal and lead revision a significant, but only minor degree of heterogeneity between studies was explained by study size. Due to possible publication bias literature-based assessments may underrate actual complication rates.

## Supporting information

S1 Table PRISMA_checklist. (DOC)

S2 Table Cohort analysis of adverse events related to DBS surgery or implanted hardware. (DOCX)

S1 Fig PRISMA flowchart. (TIF)

S2 Fig Forest plots indicating rates and 95% confidence intervals for intracranial AEs. Data from our institution (UKE Hamburg) have not been included into the meta-analysis.
(TIF)

S3 Fig Plotting of study size (no. of pats) vs. rates for intracranial AEs (y axis). The blue line indicates a local polinomial regression fitting (loess estimator), and the grey shaded area indicates the 95% confidence interval.
(TIF)

S4 Fig Funnel plot analysis of intracranial AEs. The diagonal lines (dashed) indicate the expected 95% confidence intervals around the summary estimate. According to the trim-and-fill method 29 studies were added (open circles) to adjust for funnel plot asymmetry.
(TIF)

S5 Fig Forest plots indicating rates and 95% confidence intervals for hardware removal. Data from our institution (UKE Hamburg) have not been included into the meta-analysis.
(TIF)

S6 Fig Plotting of study size (total pt.yrs) vs. rates for hardware removal (y axis). The blue line indicates a local polinomial regression fitting (loess estimator), and the grey shaded area indicates the 95% confidence interval.
(TIF)

S7 Fig Funnel plot analysis of hardware removal. The diagonal lines (dashed) indicate the expected 95% confidence intervals around the summary estimate. According to the trim-and-fill method 34 studies were added (open circles) to adjust for funnel plot asymmetry.
(TIF)

S8 Fig Forest plots indicating rates and 95% confidence intervals for lead revision. Data from our institution (UKE Hamburg) have not been included into the meta-analysis.
(TIF)

S9 Fig Plotting of study size (total pt.yrs) vs. rates for lead revision (y axis). The blue line indicates a local polinomial regression fitting (loess estimator), and the grey shaded area indicates the 95% confidence interval.
(TIF)

S10 Fig Funnel plot analysis of lead revision. The diagonal lines (dashed) indicate the expected 95% confidence intervals around the summary estimate. According to the trim-and-fill method 18 studies were added (open circles) to adjust for funnel plot asymmetry.
(TIF)
