# Supplementary material for: Towards unambiguous reporting of complications related to deep brain stimulation surgery: A retrospective single-center analysis and systematic review of the literature
Source: PLoS One. 2018 Aug 2;13(8):e0198529. doi: 10.1371/journal.pone.0198529 (PMC6071984; doi:10.1371/journal.pone.0198529)
Supplement: S2 Table — (DOCX) [file pone.0198529.s002.docx]

**S1 Table**.  **Cohort analysis of adverse events related to DBS surgery or implanted hardware**

**AE (n= # of AE) Target CTC Interval Comments**

**_________________________________________________________________________________________________________________________________**

Hemorrhage (n= 2) STN 2 – age 65; reversible moderate cognitive deficits, aspirin d/c, CAD, stenting

GPI 2 – age 75; reversible arm 'weakness'*, dysphasia, gait disturb., aspirin d/c, CAD, stenting

Hardware removal (n= 8) STN 3 3 mo IPG explanted, risk factor: diabetes mell.; reimplanted

STN 3 10 mo complete removal; no reimplantation requested despite good clinical effect

STN 3 22 mo explantation of IPG, reimplanted twice (4 procedures)

STN 3 19 mo IPG revision (submusc. placement; 19 mo); non-purulent ulceration (34 mo); reimplanted

Wound complications (n= 4) STN 3 3 wk local revision of frontal wound

GPI 3 4 mo retroauricular debridement

GPI 3 2 wk local revision at IPG site

Cm/Pf 3 3 wk local revision at IPG site

Lead revision (n= 1) STN 3 1 wk electrode replacement b/o faulty impedance, fixation plate suspicious

Device-related other (n=8) STN 3 66 mo extension wire replaced b/o fracture after repeated falls

STN 3 59 mo submuscular repositioning of IPG to prevent ulceration after 59 and 80 months

STN 3 54 mo refixation of IPG requested by patient

STN 3 5 mo discomfort b/o cicatricial traction along extension wire with revision

GPI 3 7 mo repositioning of slipped extension wire behind IPG to prevent erosion

STN 3 4 mo repositioning of slipped extension wire behind IPG to prevent erosion

GPI 3 10 mo untwisting of extension wires and refixation of IPG

**_________________________________________________________________________________________________________________________________**

A total of 23 postoperative AE related to surgery or the implanted devices occurred in 18 of the 123 patients investigated (14.6%). All AEs were rated as SAE. Each row in the table represents an individual patient. The total number of AEs (specified in the left column) exceeds the number of affected patients as, for example, infections may have involved more than one surgical procedure. The interval from initial DBS surgery is indicated in weeks (wk) or months (mo); *, 'weakness', initiation of movements was disturbed by ICH, however, muscle strength after innervation was normal; aspirin d/c, aspirin was discontinued one week prior to surgery. CTC, Common Terminology Criteria; IPG, impulse generator; ICH, intracerebral and intracranial hemorrhage; STN, subthalamic nucleus; GPI, globus pallidus internus; VIM, nucleus ventralis intermedius thalami; Cm/Pf, centre médian-parafascicular nuclei of thalamus; CAD, coronary artery disease
