# Supplementary figures and images for: Towards unambiguous reporting of complications related to deep brain stimulation surgery: A retrospective single-center analysis and systematic review of the literature
Source: PLoS One. 2018 Aug 2;13(8):e0198529. doi: 10.1371/journal.pone.0198529 (PMC6071984; doi:10.1371/journal.pone.0198529)

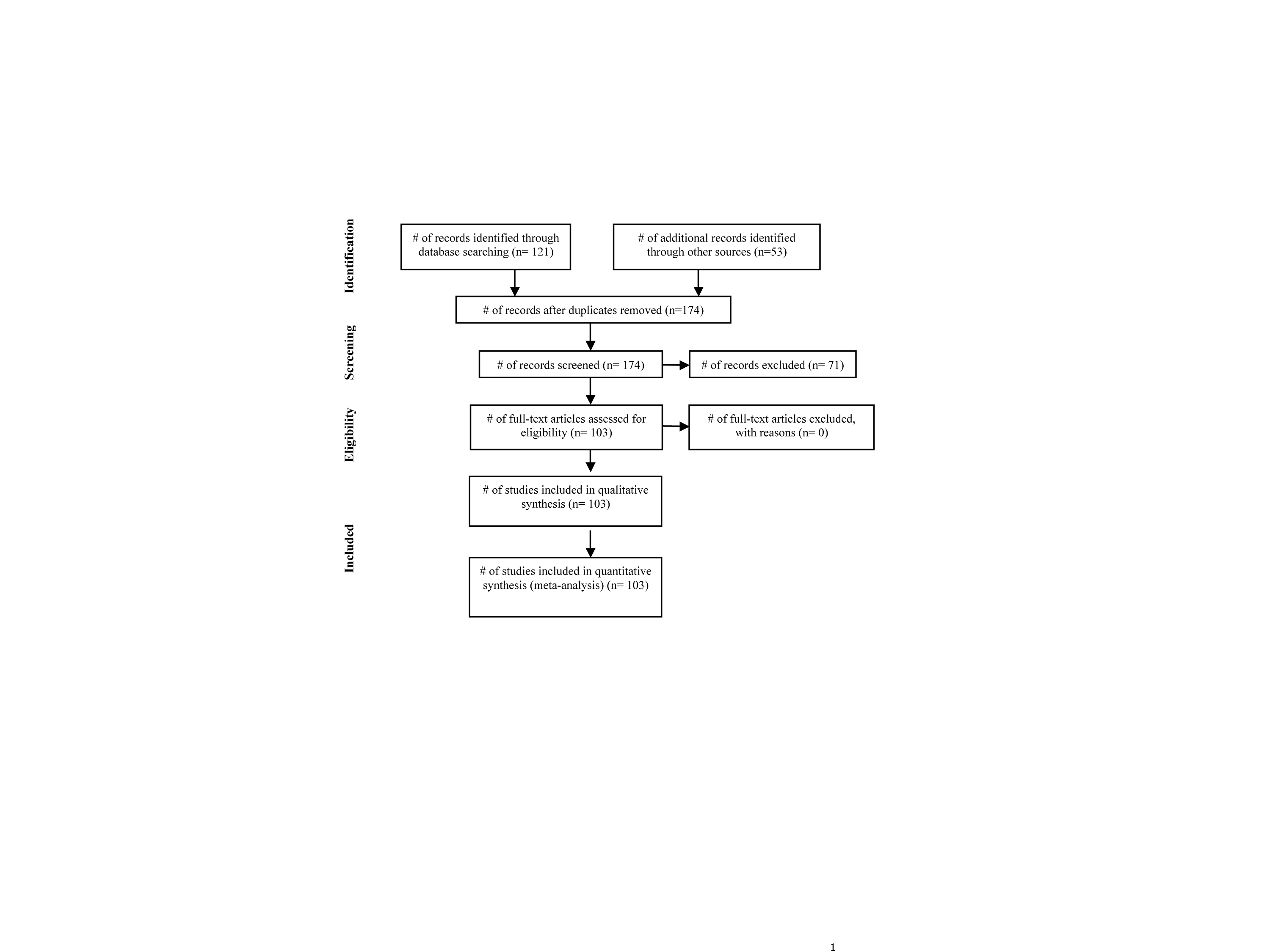

Supplement: S1 Fig — (TIF) [file pone.0198529.s003.tif]

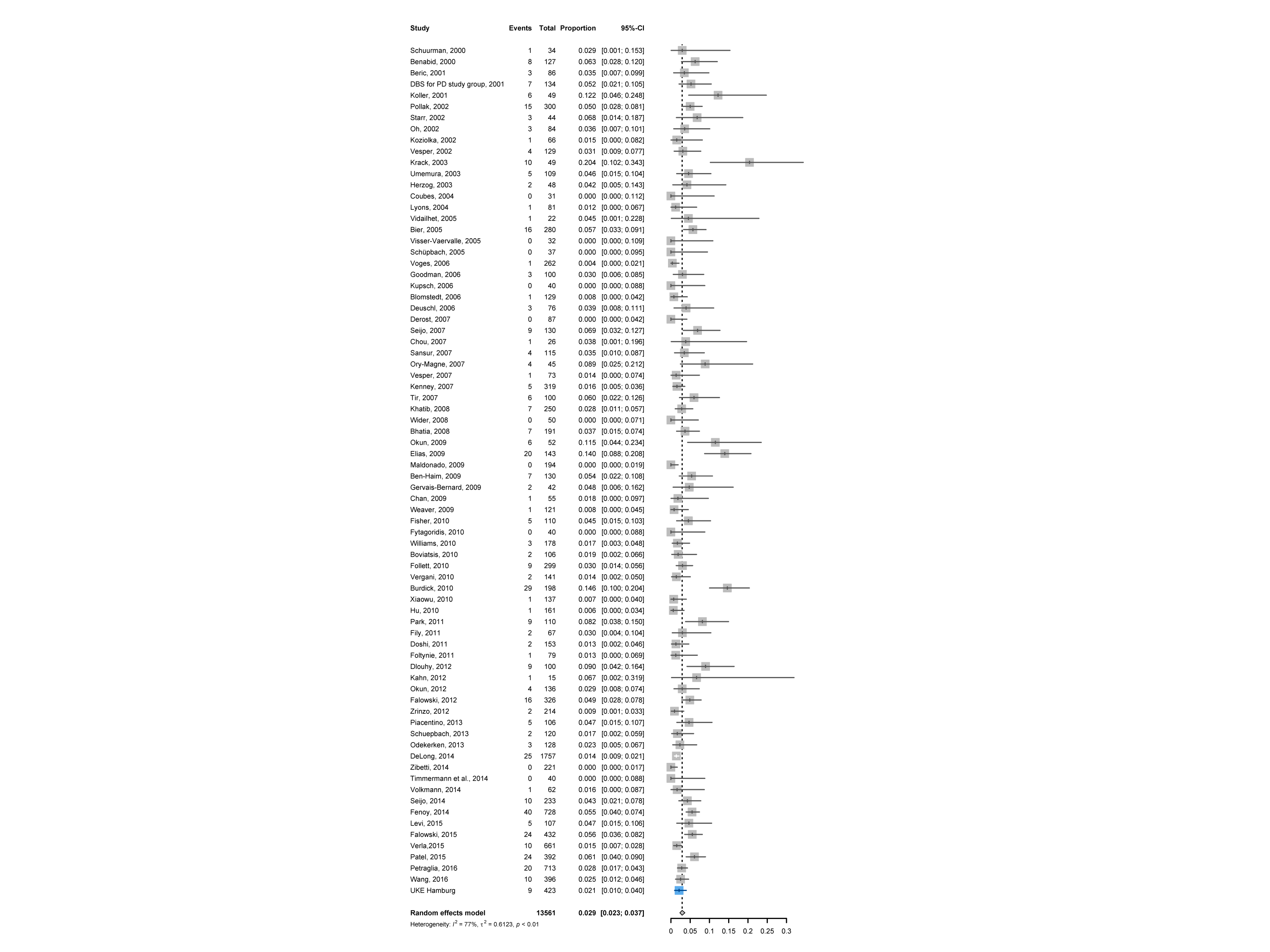

Supplement: S2 Fig — Data from our institution (UKE Hamburg) have not been included into the meta-analysis. (TIF) [file pone.0198529.s004.tif]

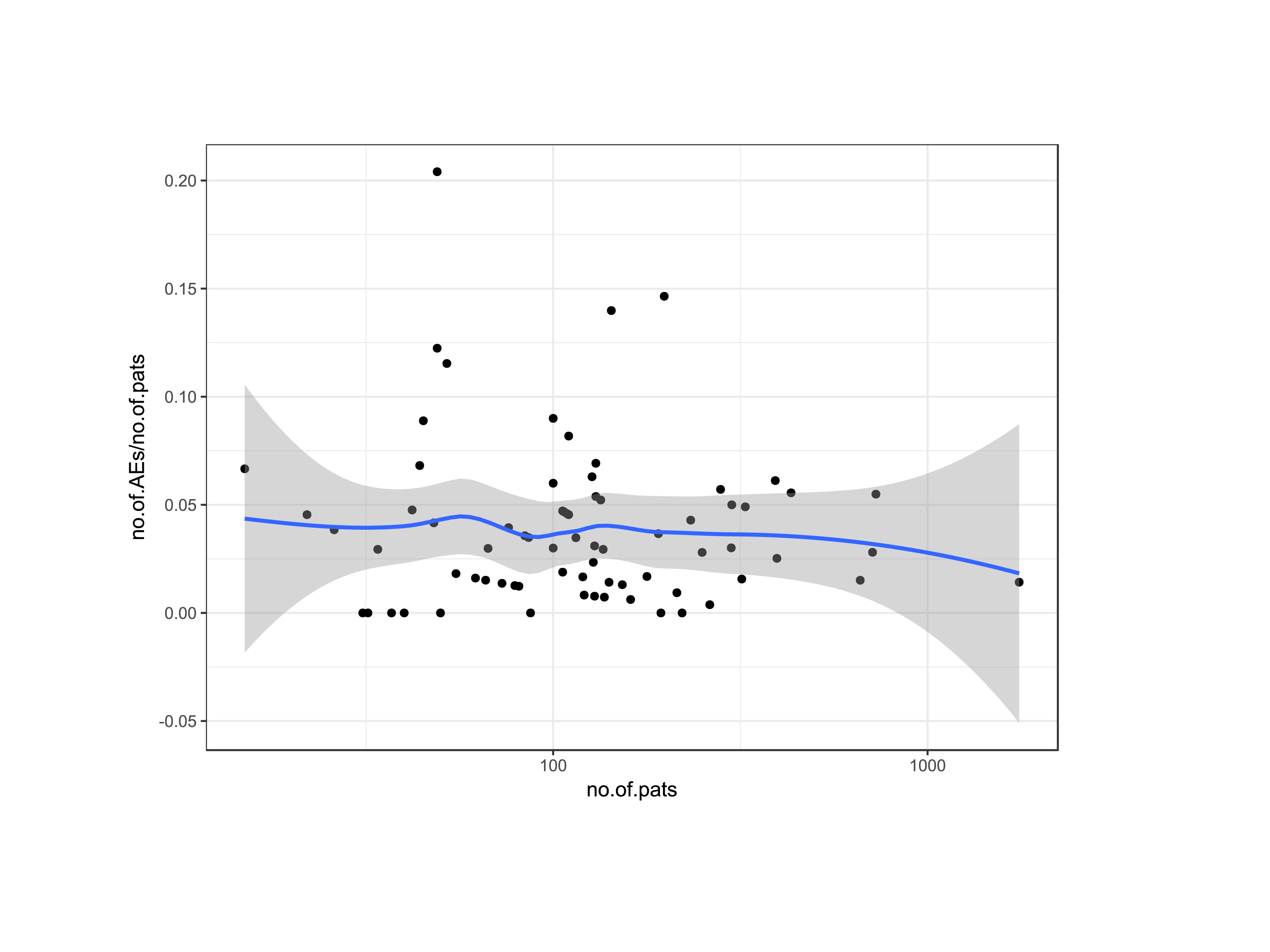

Supplement: S3 Fig — The blue line indicates a local polinomial regression fitting (loess estimator), and the grey shaded area indicates the 95% confidence interval. (TIF) [file pone.0198529.s005.tif]

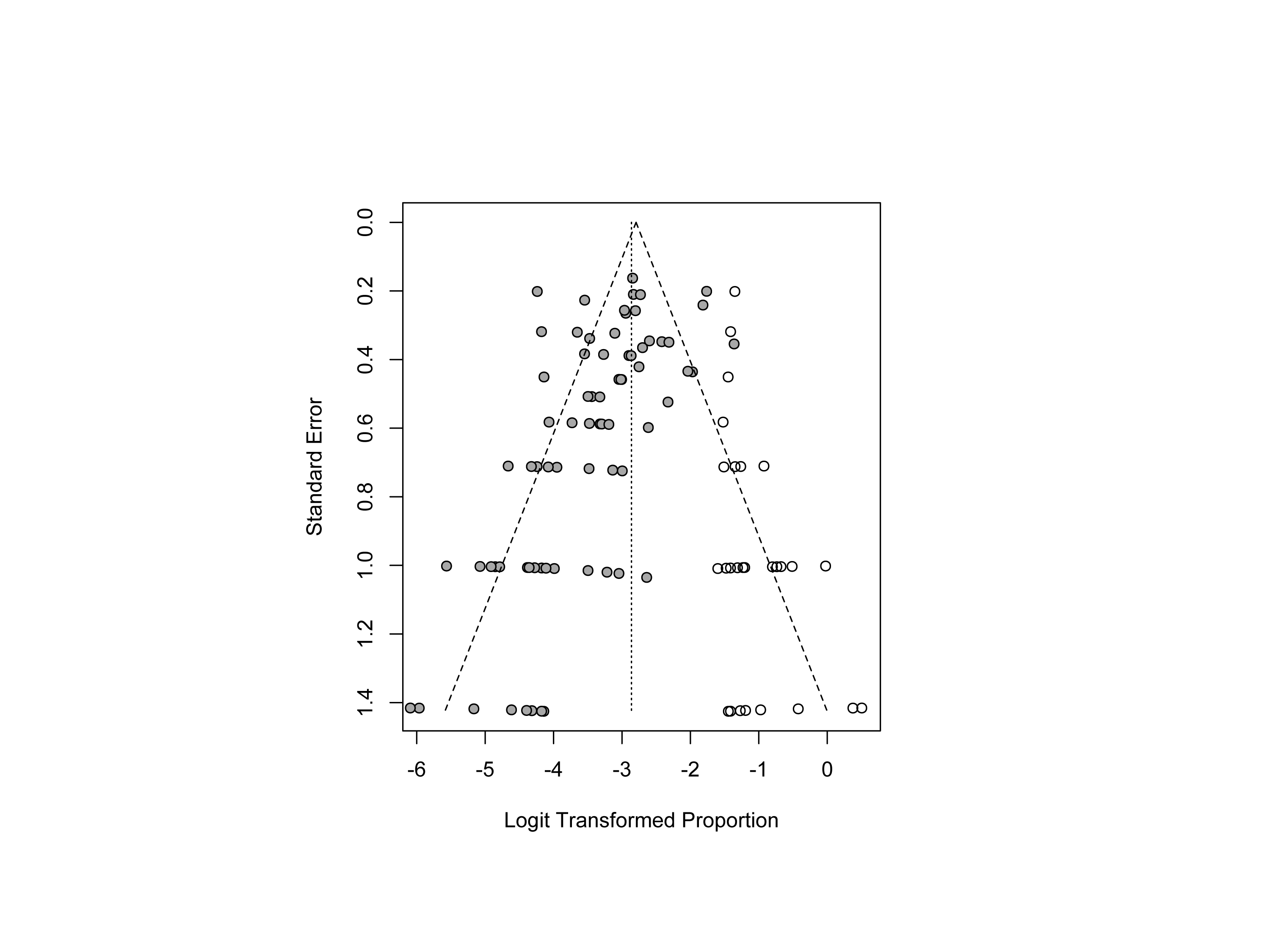

Supplement: S4 Fig — The diagonal lines (dashed) indicate the expected 95% confidence intervals around the summary estimate. According to the trim-and-fill method 29 studies were added (open circles) to adjust for funnel plot asymmetry. (TIF) [file pone.0198529.s006.tif]

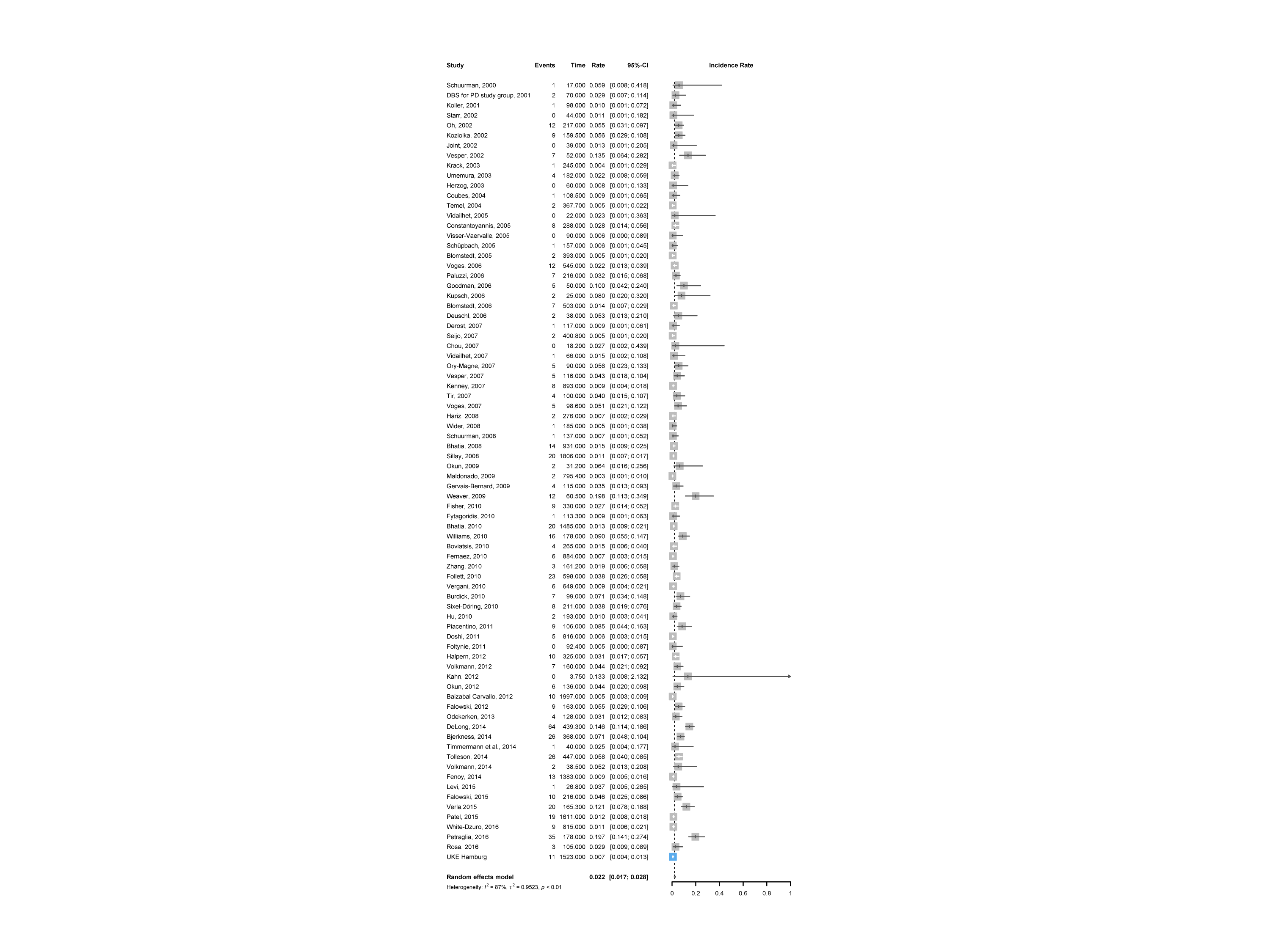

Supplement: S5 Fig — Data from our institution (UKE Hamburg) have not been included into the meta-analysis. (TIF) [file pone.0198529.s007.tif]

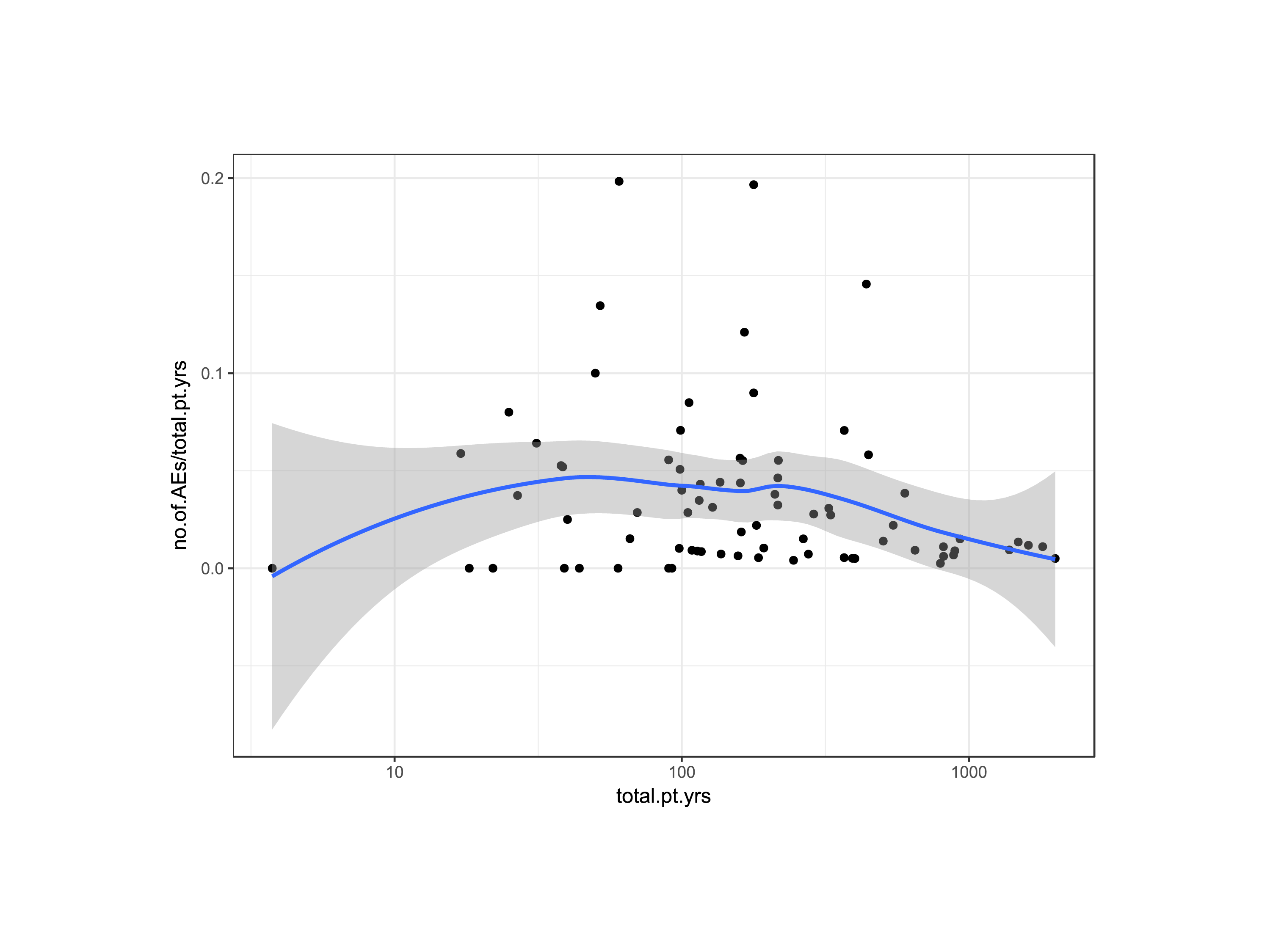

Supplement: S6 Fig — The blue line indicates a local polinomial regression fitting (loess estimator), and the grey shaded area indicates the 95% confidence interval. (TIF) [file pone.0198529.s008.tif]

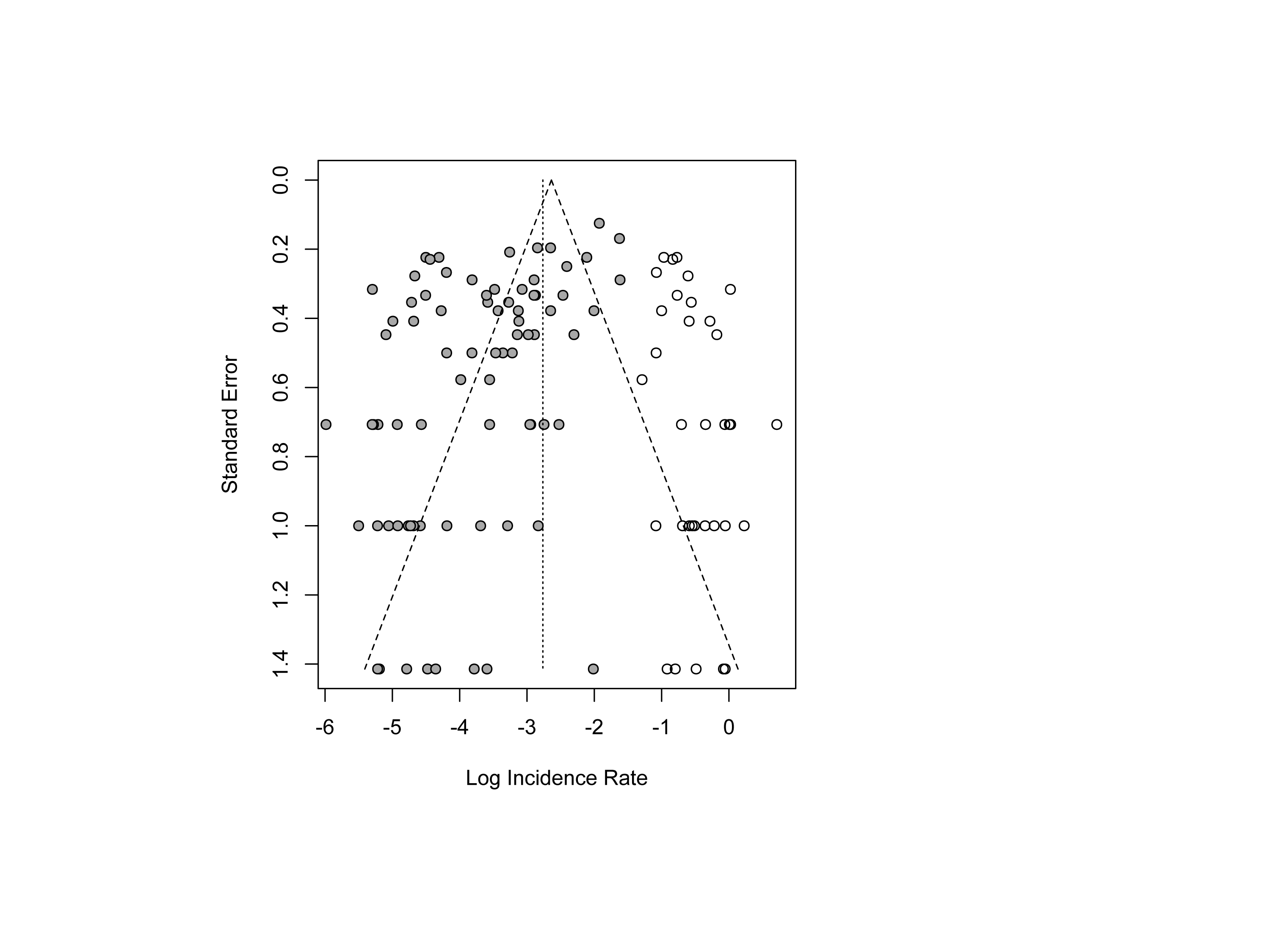

Supplement: S7 Fig — The diagonal lines (dashed) indicate the expected 95% confidence intervals around the summary estimate. According to the trim-and-fill method 34 studies were added (open circles) to adjust for funnel plot asymmetry. (TIF) [file pone.0198529.s009.tif]

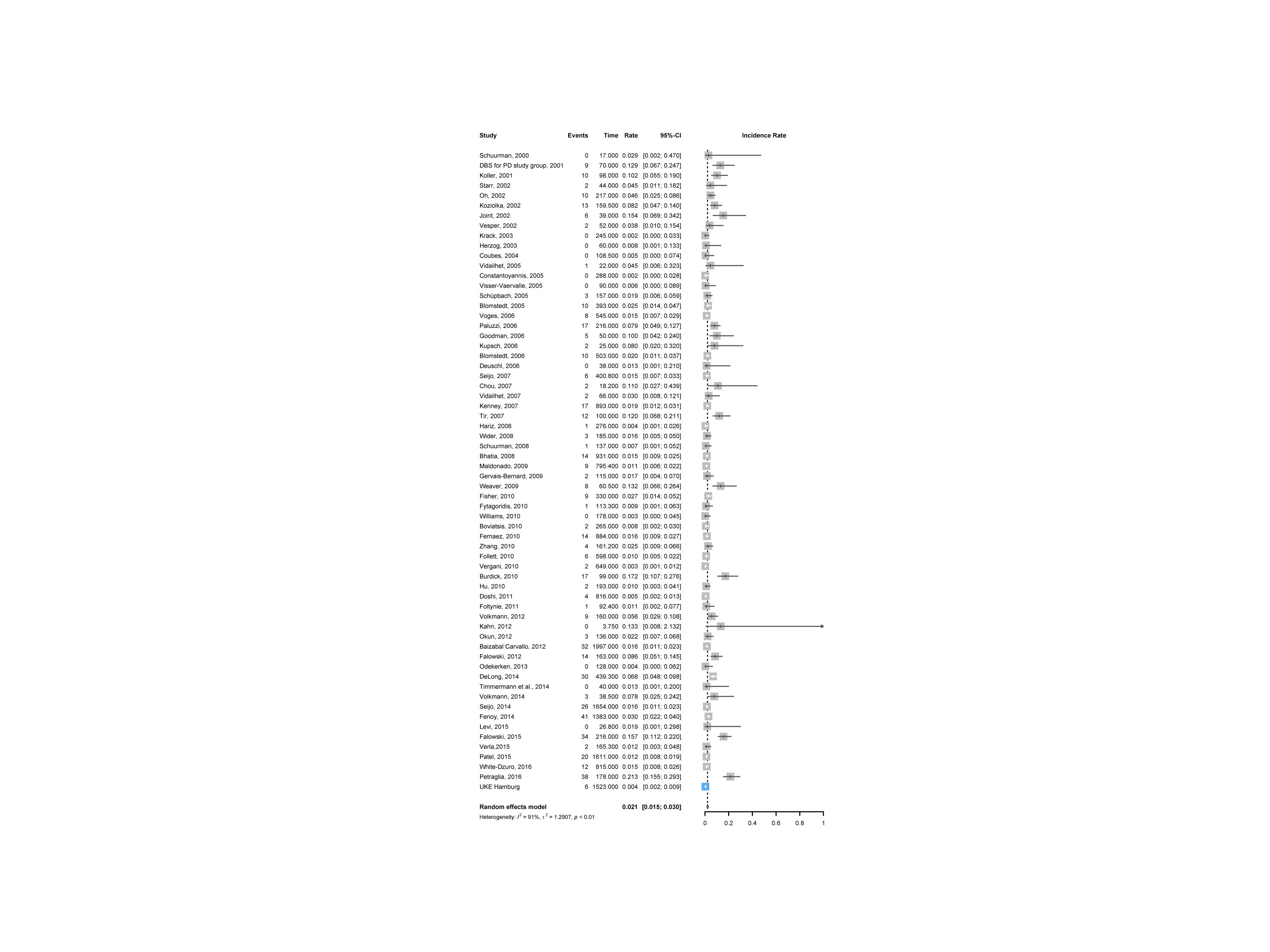

Supplement: S8 Fig — Data from our institution (UKE Hamburg) have not been included into the meta-analysis. (TIF) [file pone.0198529.s010.tif]

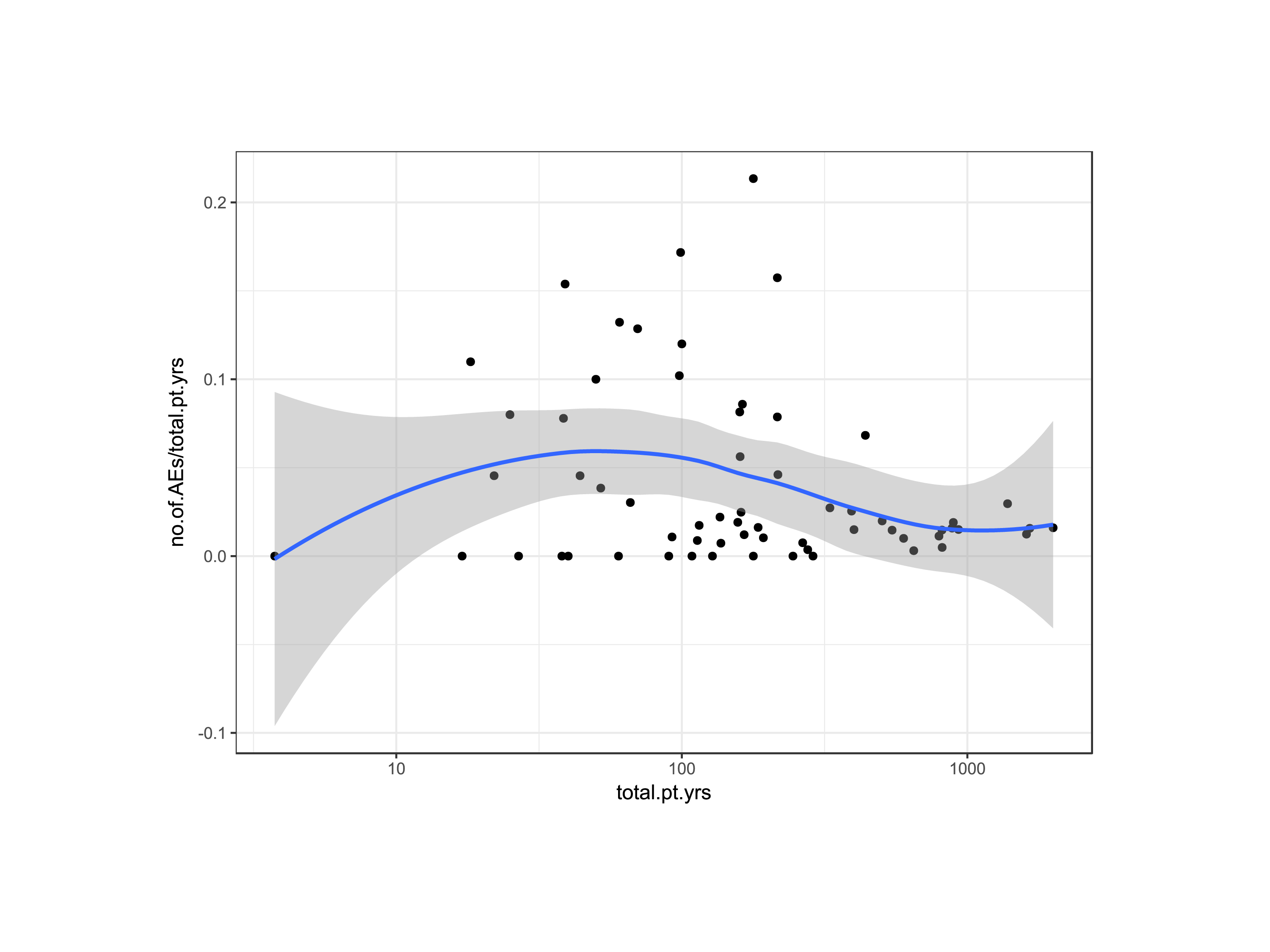

Supplement: S9 Fig — The blue line indicates a local polinomial regression fitting (loess estimator), and the grey shaded area indicates the 95% confidence interval. (TIF) [file pone.0198529.s011.tif]

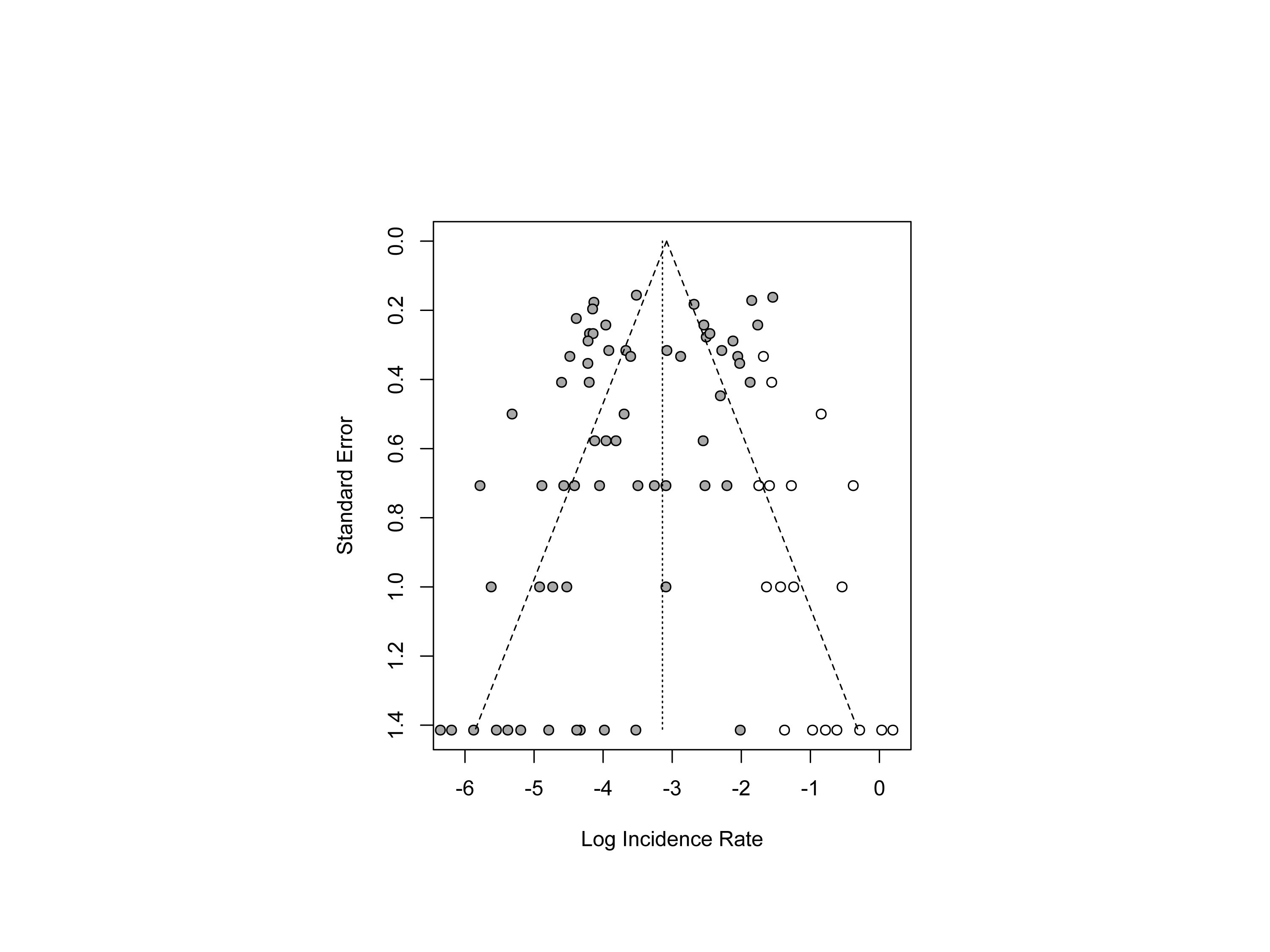

Supplement: S10 Fig — The diagonal lines (dashed) indicate the expected 95% confidence intervals around the summary estimate. According to the trim-and-fill method 18 studies were added (open circles) to adjust for funnel plot asymmetry. (TIF) [file pone.0198529.s012.tif]
